# DJ-1 maintains energy and glucose homeostasis by regulating the function of brown adipose tissue

**DOI:** 10.1038/celldisc.2016.54

**Published:** 2017-02-14

**Authors:** Rong Wu, Xiao-meng Liu, Jian-guang Sun, Hong Chen, Jun Ma, Meng Dong, Shengyi Peng, Ji-qiu Wang, Jian-qing Ding, Dong-hao Li, John R Speakman, Guang Ning, Wanzhu Jin, Zengqiang Yuan

**Affiliations:** 1 State Key Laboratory of Brain and Cognitive Sciences, Institute of Biophysics, Chinese Academy of Sciences, Beijing, China; 2 College of Life Sciences, University of Chinese Academy of Sciences, Beijing, China; 3 Key Laboratory of Animal Ecology and Conservation Biology, Institute of Zoology, Chinese Academy of Sciences, Beijing, China; 4 College of life science and agronomy, Zhoukou Normal University, Zhoukou, China; 5 Sino-Danish Center Neuroscience Program, University of Chinese Academy of Sciences, Beijing, China; 6 Ruijin Hospital Affiliated to Shanghai Jiaotong University School of Medicine, Shanghai, China; 7 Key Laboratory of Natural Resources of Changbai Mountain and Functional Molecules, Ministry of Education, Yanbian University, Yanji, China; 8 State Key Laboratory of Molecular Developmental Biology (Beijing), Institute of Genetics and Developmental Biology, Chinese Academy of Sciences, Beijing, China; 9 Institute of Biological and Environmental Sciences, University of Aberdeen, Aberdeen, UK

**Keywords:** BAT, DJ-1, obesity, ubiquitination, Ucp1

## Abstract

DJ-1 protein is involved in multiple physiological processes, including Parkinson’s disease. However, the role of DJ-1 in the metabolism is largely unknown. Here we found that DJ-1 maintained energy balance and glucose homeostasis* *via regulating brown adipose tissue (BAT) activity. DJ-1-deficient mice reduced body mass, increased energy expenditure and improved insulin sensitivity. DJ-1 deletion also resisted high-fat-diet (HFD) induced obesity and insulin resistance. Accordingly, DJ-1 transgene triggered autonomous obesity and glucose intolerance. Further BAT transplantation experiments clarified DJ-1 regulates energy and glucose homeostasis by modulating BAT function. Mechanistically, we found that DJ-1 promoted PTEN proteasomal degradation via an E3 ligase, mind bomb-2 (Mib2), which led to Akt activation and inhibited FoxO1-dependent Ucp1 (Uncoupling protein-1) expression in BAT. Consistently, ablation of Akt1 mitigated the obesity and BAT dysfunction induced by DJ-1 transgene. These findings define a new biological role of DJ-1 protein in regulating BAT function, with an implication of the therapeutic target in the treatment of metabolic disorders.

## Introduction

Obesity occurs when energy intake exceeds energy expenditure [1]. Obesity has reached epidemic proportions worldwide and is accompanied by a series of metabolic diseases including type II diabetes, hepatic steatosis and cardiovascular diseases [2]. Increasing energy expenditure, especially promoting brown adipose tissue (BAT) thermogenesis [1], has received considerably more attention than inhibiting energy intake and/or absorption as an effective anti-obesity treatment. It has been shown that BAT activity is inversely correlated with body mass index (BMI) and the percentage of body fat [3, 4]. Recently, we and other groups have shown that BAT transplantation can improve whole body energy metabolism and glucose homeostasis [5–7].

DJ-1 has been implicated in reproduction [8–10], androgen receptor signaling [11–13] and tumorigenesis [14, 15]. Recent evidence indicates that mutations in the DJ-1 gene are associated with early-onset Parkinsonism [16]. DJ-1 could also be connected with two other Parkinson disease-related genes; *Parkin* and Pten-induced putative kinase 1 (*PINK1*) to form the E3 ligase complex, thereby promoting the degradation of unfolded proteins and Parkinsonism [17]. Interestingly, *Parkin* and *PINK1* are reported to be involved in the regulation of lipid metabolism [18, 19].

DJ-1-mediated PI3K/Akt activation has been reported to occur in neuronal protection and tumorigenesis [15, 20, 21]. PTEN (phosphatase and tensin homologue deleted on chromosome 10), a putative phosphatase, has a dominant inhibitory role in PI3K/Akt signaling [22]. PTEN transgenic (Tg) mice have reduced body weight, elevated energy expenditure and insulin sensitivity [23, 24]. The activity of BAT is activated in PTEN Tg mice, but impaired in Myf5-cre-mediated PTEN knockout (KO) mice [25]. These findings indicate that the PTEN-Akt cascade participates in the regulation of insulin sensitivity and BAT function. However, whether and how DJ-1 is involved in energy and glucose homeostasis and BAT function are largely unknown.

## Results

### DJ-1 KO mice have reduced adiposity, increased energy expenditure and insulin sensitivity

**Figure 1:**
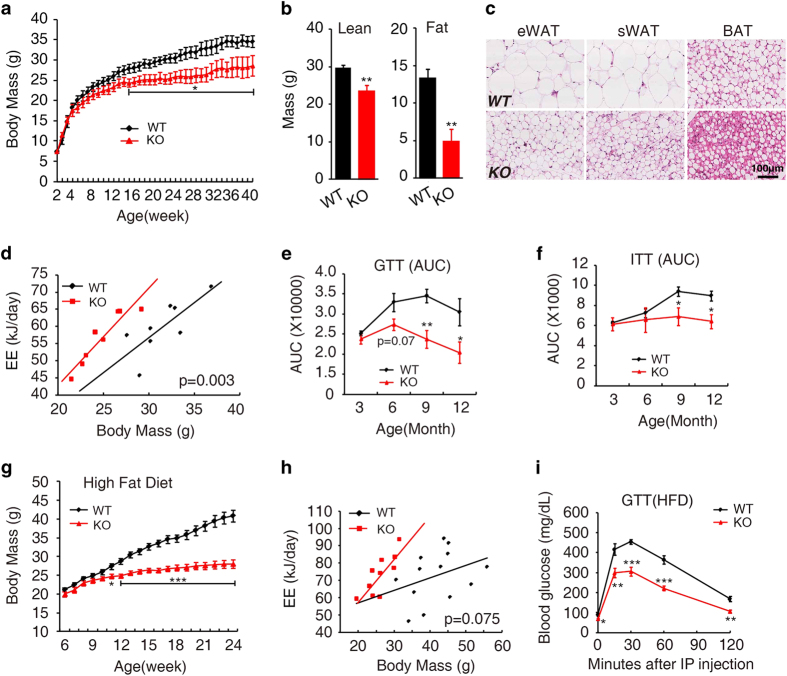
Reduced body mass, increased energy expenditure and improved insulin sensitivity in DJ-1 knockout mice during aging and high-fat diet. (**a**) Body mass of wild-type (WT) and DJ-1 knockout male mice (KO) fed on a chow diet for 40 weeks (WT, *n*=10; KO, *n*=10). (**b**) Body lean and fat mass of 12-month-old mice measured by MRI (WT, *n*=7; KO, *n*=6). (**c**) Histology of adipose tissue from 12-month-old mice. eWAT, epididymal white adipose tissue; sWAT, subcutaneous white adipose tissue; BAT, brown adipose tissue. (**d**) Statistical analysis energy expenditure (EE) by General Linear Model (GLM) and ANCOVA analysis of the 6-month-old male DJ-1 KO and WT mice (*n*=8 per genotype). (**e**, **f**) Quantification of the area under the curve (AUC) of GTT (**e**) and ITT (**f**) indicated age (*n*=6 per genotype). (**g**) Body mass of mice that were fed with a high-fat diet (HFD) (WT, *n*=14; KO, *n*=13). (**h**) Statistical analysis by General Linear Model (GLM) and ANCOVA analysis of the HFD induced indicated genotype mice energy expenditure (EE) (WT, *n*=13; KO, *n*=10). (**i**) GTT was performed in HFD-fed mice with intraperitoneal injection (IP) 2 g kg^−1^ glucose after fasting 16 h (WT, *n*=6; KO, *n*=8).

No developmental abnormalities were observed in DJ-1 KO mice compared with wild-type (WT) mice. However, we found a significant reduction in body mass in DJ-1 KO mice compared with their WT counterparts from the maturity-onset stage (age 16 weeks) onwards (Figure 1a). Magnetic resonance imaging (MRI) showed an increase in the percentage of lean mass and a reduction in the percentage of body fat in DJ-1 KO mice (Figure 1b). Further examination showed that the reduction in the percentage of body fat in DJ-1 KO mice was mainly due to a reduction in the mass of epididymal white adipose tissue (eWAT), subcutaneous white adipose tissue (sWAT) and brown adipose tissue (BAT), but not other tissues, without lower free fatty acid (FFA) in plasma (Supplementary Figure S1A–D). Consistent with MRI results, histological analysis revealed that lipid droplets in adipose tissues from DJ-1 KO mice were smaller compared with those in WT mice (Figure 1c). These results indicate that deletion of DJ-1 specifically affects adipose tissue composition.

Changes in adiposity are often accompanied with alterations in energy balance. We next examined whether there was any change in energy metabolism in DJ-1 KO mice. Interestingly, we found a significant increase in energy expenditure in DJ-1 KO mice compared with WT mice (Figure 1d), even though there was no significant difference in respiratory quotients (Supplementary Figure S1E), food intake (Supplementary Figure S1F) and physical activity (Supplementary Figure S1G) between two groups. These results suggest that reduced adiposity in DJ-1 KO mice might be due to an increase in energy expenditure.

Alterations in adiposity often affect insulin sensitivity. To clarify this point, we performed glucose tolerance tests (GTT) in WT and DJ-1 KO mice. Consistent with body mass results, there was no change in glucose tolerance in 3-month-old mice between the two groups (Figure 1e and Supplementary Figure S1H). However, glucose tolerance was significantly improved in aged DJ-1 KO mice compared with WT mice (Figure 1e and Supplementary Figure S1I–K). The insulin tolerance tests (ITT) showed the similar results (Figure 1f and Supplementary Figure S1L–O). These data suggest that progressive improvement of insulin sensitivity in DJ-1 KO mice was due to a reduction in adiposity.

The difference in adiposity between DJ-1 KO and WT mice was further enhanced in the HFD (high-fat diet) model (Figure 1g). After 5-week of HFD, body mass gain was significantly reduced in DJ-1 KO mice (*P*<0.05) and this difference was much more evident in the long-term HFD treatment (*P*<0.001; Figure 1g). MRI analysis showed that lean mass and body fat mass decreased in DJ-1 KO mice (Supplementary Figure S2A). In particular, the mass of eWAT, sWAT and BAT were markedly reduced in DJ-1 KO mice (Supplementary Figure S2B). Furthermore, histological analysis showed a marked decrease in HFD-induced lipid accumulation in both adipose tissue and the liver in DJ-1 KO mice (Supplementary Figure S2C). Consistent with results from chow diet experiments, energy expenditure was significantly increased in DJ-1 KO mice (Figure 1h and Supplementary Figure S2D), and led to improved insulin sensitivity in DJ-1 KO mice during HFD (Figure 1i). Taken together, these results indicate that the increased energy expenditure significantly reduced adiposity and improved insulin sensitivity in DJ-1 KO mice.

### Overexpression of DJ-1 triggers obesity and glucose intolerance

Significant reduction in adiposity and improvement of energy metabolism in DJ-1 KO mice prompted us to determine whether overexpression of DJ-1 is sufficient to initiate obesity. DJ-1 Tg (Transgenic) mice were generated by transgenically overexpressing DJ-1 (Supplementary Figure S3A). Transgenic DJ-1 expression was observed in the various tissues (Supplementary Figure S3B).

**Figure 2:**
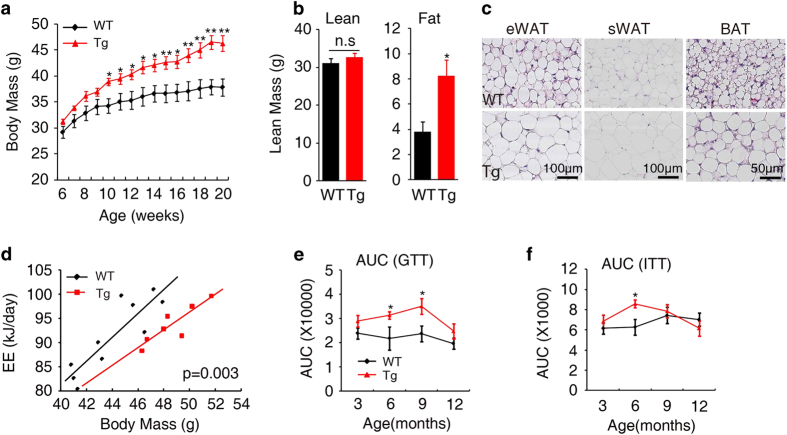
The DJ-1 transgene increases body mass gain and glucose intolerance. (**a**) Body mass cure of wild-type (WT) and DJ-1 transgenic mice (Tg, Line 13 CD-1 background) fed on a chow diet (WT, *n*=9; Tg, *n*=10). (**b**) Body lean and fat mass measured by MRI (WT, *n*=6; Tg, *n*=6). (**c**) HE staining of eWAT, sWAT and BAT from 6-month-old mice. (**d**) Statistical analysis by General Linear Model (GLM) and ANCOVA analysis of the DJ-1 Tg and WT mice energy expenditure (EE) (WT, *n*=10; Tg, *n*=7). (**e**) and (**f**) Quantification on the area under the curve (AUC) of GTT (**e**) and ITT (**f**) indicated age (*n*=6–12).

DJ-1 Tg mice showed no developmental abnormalities. The body mass of DJ-1 Tg mice increased significantly from 10-week age (Figure 2a). MRI analysis showed that DJ-1 Tg mice had increased fat mass, although as there is no significant change in lean mass (Figure 2b). Consistently, there was a marked increase in adipose tissue mass but not other tissues, with similar concentrations of free fatty acid (Supplementary Figure S3C and D). In addition, histological analysis revealed that lipid droplets in adipose tissues from DJ-1 Tg mice were larger than those in WT mice (Figure 2c).

There was a significant reduction in energy expenditure and increased respiratory quotients in DJ-1 Tg mice (Figure 2d and Supplementary Figure S3E). The defect in energy expenditure was not due to either food intake (Supplementary Figure S3F) or physical activity (Supplementary Figure S3G) in DJ-1 Tg mice. Additionally, we found that there is marked glucose intolerance (Figure 2e and Supplementary Figure S3H) in DJ-1 Tg mice. Insulin tolerance tests showed DJ-1 transgenic had a little effect on insulin sensitivity (Figure 2f and Supplementary Figure S3I). Taken together these results show that expression of the DJ-1 transgene is sufficient to induce obesity and glucose intolerance.

### DJ-1 regulates Ucp1 expression in brown adipocytes

**Figure 3:**
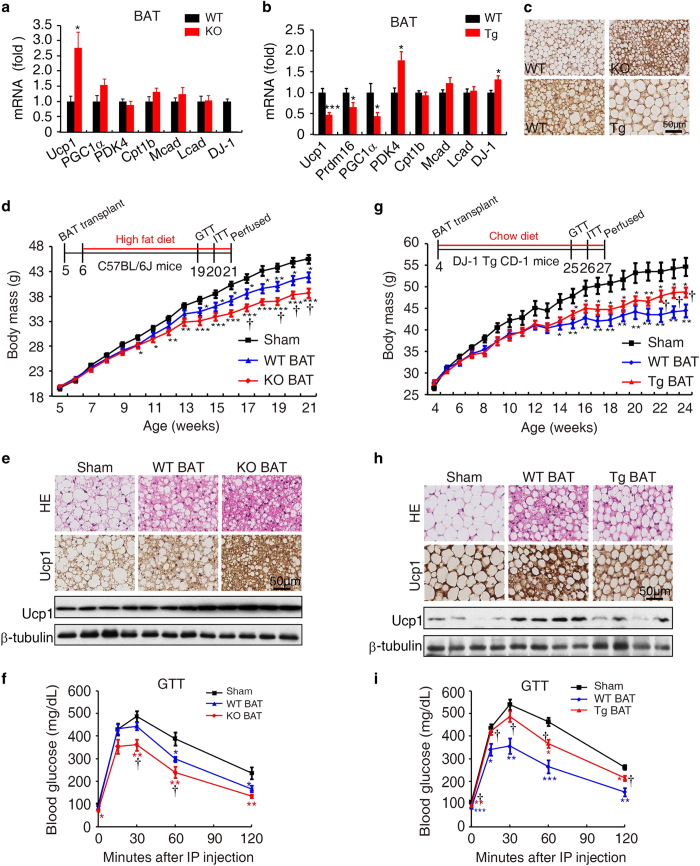
DJ-1 maintains BAT function. (**a**) Gene expression in BAT from 12-month-old WT and DJ-1 KO mice (*n*=6 per genotype). (**b**) Gene expression in BAT from 4-week-old WT and DJ-1 transgenic mice (WT, *n*=6; TG, *n*=7). (**c**) Ucp1 immunostaining of BAT from indicated genotype mice, upper panel tissues from 12-month-old mice and bottom panel tissues from 6-month old. (**d**) Body mass of mice with sham operation, BAT transplantation from wild-type (WT BAT) and DJ-1 KO (KO BAT) mice (sham, *n*=11; WT BAT, *n*=13; KO BAT, *n*=8). (**e**) HE (upper panel), Ucp1 immunostaining (middle panel) and immunoblotting (bottom panel) of endogenous BAT from sham operation, WT BAT transplantation and KO BAT transplantation mice. (**f**) GTT assays of 19-week-old and HFD-induced recipient mice (sham, WT BAT or DJ-1 KO BAT). (**g**) Body mass curve of the receiver mice of WT BAT or Tg BAT transplantation or sham (*n*=6 per group). WT BAT or Tg BAT was transplanted subcutaneously with the BAT from wild type or DJ-1 transgenic mice. (**h**) HE (upper panel), Ucp1 immunostaining (middle panel) and immunoblotting (bottom panel) of the endogenous BAT from the groups indicated. (**i**) GTT assay results for 25-week-old DJ-1 transgenic mice transplanted with BAT. (Data are shown as the mean±s.e.m. NS, non significant, *P<0.05, **P<0.01, ***P<0.001 in comparison with sham, ^†^*P*<0.05, ^††^*P*<0.01 in comparison with WT BAT, determined using the two-tailed Student’s *t*-test).

Our finding that DJ-1 KO increases energy expenditure and improves insulin sensitivity, whereas DJ-1 Tg confers the opposite phenotype, strongly suggests that DJ-1 may be involved in energy metabolism. BAT is known to consume fatty acids and glucose to contribute energy metabolism [26]. Interestingly, we found that Ucp1, specifically expressed in BAT, was increased markedly in DJ-1 KO mice, although the expression of other fatty acid oxidation-related genes was not altered (Figure 3a). In contrast, BAT-specific genes, including Ucp1, Prdm16 and PGC1α, were markedly decreased in DJ-1 Tg mice (Figure 3b). Immunohistochemical analysis further confirmed that Ucp1 protein levels increased in DJ-1 KO mice and decreased in DJ-1 transgenic mice (Figure 3c). These results indicate that DJ-1 may be involved in the regulation of Ucp1 expression.

Sympathetic outflow from ventral hypothalamus to BAT and WAT controls the expression of thermogenic genes and heat production in brown and beige fat [27]. To test whether ventral hypothalamus controls the sympathetic drive to brown fat, we measured the expression levels of genes encoding for orexigenic and anorexigenic neuropeptides in the ventral hypothalamus. Levels of Agrp, Npy, Pomc, leptin receptor (Leprb) and Signal transducer and activator of transcription 3 (Stat3) were not significant different among the genotypes (Supplementary Figure S4A and B). In addition, we examined the mRNA level of tyrosine hydroxylase (TH), a marker of sympathetic nerve, which has no significant change in BAT from DJ-1 KO or DJ-1 Tg mice compared with their wild-type controls (Supplementary Figure S4C). These results indicated thermogenic Ucp1 expression was not regulated by sympathetic outflow in DJ-1 KO or Tg mice. In addition, we explored whether DJ-1 knockout altered the lipolysis-related epinephrine and norepinephrine signaling in WAT and BAT. We found that DJ-1 KO had no significant effect in the phosphorylation of hormone sensitive lipase (HSL), which is a putative target of epinephrine and norepinephrine signaling, in both BAT and WAT (Supplementary Figure S4D).

To investigate whether DJ-1 is involved in BAT differentiation, primary brown adipose precursors were isolated from newborn WT and DJ-1 KO mice followed by *in vitro* BAT differentiation assays. There was a significant increase of pre-adipocyte differentiation ability in DJ-1 KO BAT (Supplementary Figure S4E). The expression of BAT marker genes, including Ucp1 and Prdm16, were markedly increased in differentiated BAT cells from DJ-1 KO mice (Supplementary Figure S4F). Accordingly, DJ-1 transgene confers a significant reduction of pre-adipocyte differentiation ability (Supplementary Figure S4E). The expression of BAT marker genes was markedly decreased in differentiated BAT cells from DJ-1 Tg mice (Supplementary Figure S4G). Taken together, DJ-1 regulates Ucp1 expression in cell autonomous manner.

### DJ-1 is involved in the maintenance of BAT functional integrity

Recently, BAT transplantation has been shown to improve energy expenditure and glucose homeostasis [6, 7]. We next investigated whether DJ-1 is involved in BAT functional integrity through BAT transplantation experiments. WT mice were subcutaneously transplanted with BAT from WT or DJ-1 KO mice and followed with HFD treatment. Compared with transplantation of WT BAT, transplantation of DJ-1 KO BAT significantly ameliorated HFD-induced body mass gain (Figure 3d). Consistent with our recent study [7], fat and liver mass were significantly decreased after WT or DJ-1 KO BAT transplantation (Supplementary Figure S5A). The size of endogenous brown adipocytes was smaller in mice transplanted with DJ-1 KO BAT than in those transplanted with WT BAT or sham operated mice upon HFD treatment (Figure 3e), a phenomenon often observed in active BAT. There was no difference in the size of adipocytes in eWAT and sWAT (Supplementary Figure S5B). In parallel, transplantation of DJ-1 KO BAT markedly reversed HFD-induced hepatic steatosis compared with the sham control, although WT BAT transplantation had an intermediate rescue effect (Supplementary Figure S5B). Consistent with reports that exogenous BAT can enhance the function of endogenous BAT [7, 28], transplantation of DJ-1 KO BAT significantly induced Ucp1 expression in endogenous BAT, as determined by immunohistochemistry and Western blotting (Figure 3e). Further GTT and ITT analysis showed that there are significant improvements in glucose tolerance and insulin sensitivity after BAT transplantation (Figure 3f and Supplementary Figure S5C).

In contrast to transplantation of DJ-1 KO BAT, we found DJ-1 Tg BAT transplantation failed to improve the functional integrity of endogenous BAT (Figure 3g and h). DJ-1 Tg mice were used as recipients, and subcutaneously transplanted with BAT from WT or DJ-1 Tg mice. WT BAT transplantation significantly reduced obesity compared with DJ-1 Tg BAT (Figure 3g, Supplementary Figure S5D and E). Immunohistochemistry and western blotting showed that transplanting WT BAT significantly increased endogenous BAT Ucp1 expression. However, transplanting DJ-1 Tg BAT significantly reduced Ucp1 expression (Figure 3h). In addition, we observed that WT BAT transplantation markedly improved glucose homeostasis compared with transplantation of DJ-1 Tg BAT (Figure 3i and Supplementary Figure S5F). Taken together, these results indicate that DJ-1 is important for BAT functional integrity.

### DJ-1 regulates Ucp1 expression through PTEN-Akt-FoxO1 signaling cascade

**Figure 4:**
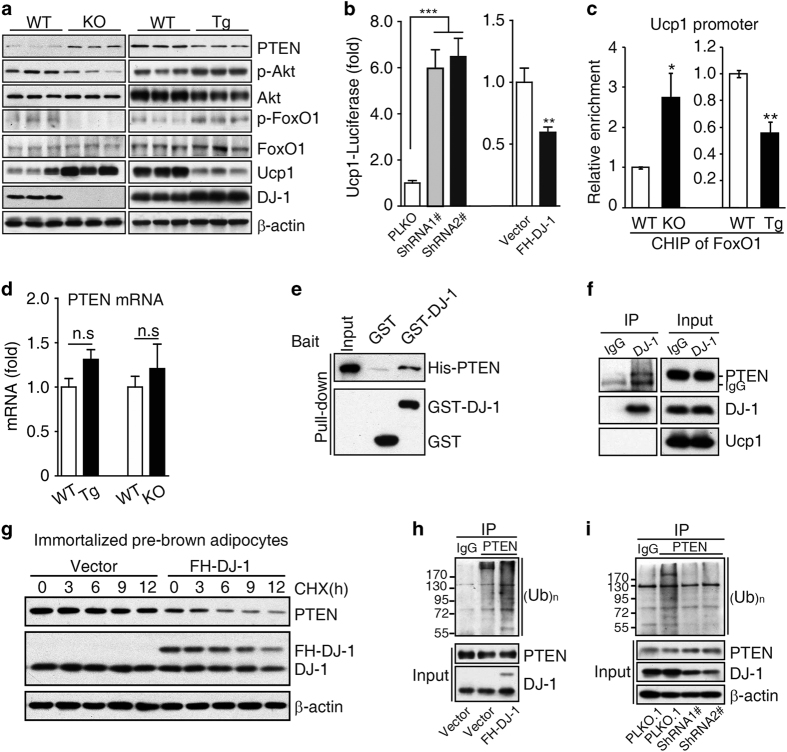
DJ-1 regulates Ucp1 expression by promoting PTEN degradation in brown adipocytes. (**a**) Lysates of BAT from 12-month-old indicated genotype mice and subjected to immunoblotting with the indicated antibodies. (**b**) DJ-1 was stably knocked down by lentivirus shRNA or overexpressed with retrovirus in immortalized brown adipose precursors. Ucp1-luciferase reporter activity was examined (Student’s *t*-test, **P<0.01, ***P<0.001, *n*>4). (**c**) ChIP analysis of FoxO1 on the Ucp1 promoter in BAT from indicated genotype mice (*n*=3 per group, Student’s *t*-test, *P<0.05, **P<0.01). (**d**) PTEN mRNA levels in BAT from 4-week-old DJ-1 transgenic mice and 12-month-old DJ-1 KO mice and their controls (*n*=6–7 per group). (**e**) GST-DJ-1 immobilized on glutathione-Sepharose beads pulls down recombinant His tagged PTEN. Pull downs were immunoblotted with anti-PTEN. (**f**) Lysates of BAT were immunoprecipitated with DJ-1 antibody followed by immunoblotting with PTEN antibody. (**g**) Cells stably transfected with FH (Flag and HA) tagged DJ-1 or the control vector were treated with 100 μg ml^−1^ CHX for the number of hours indicated. Equal amounts of total protein lysates were immunoblotted with PTEN or β-actin antibody. (**h**, **i**) Endogenous PTEN ubiqutination was examined in brown adipocytes with overexpression FH tagged DJ-1 (**h**) or lentivirus vector PLKO.1 vector-mediated DJ-1 knockdown (**i**) after MG132 treatment 8 h.

We next explored the molecular mechanism underlying DJ-1 regulation of BAT functional integrity. A previous report demonstrated that the PTEN-Akt-FoxO1 pathway regulates Ucp1 expression in BAT [23]. DJ-1 has also been shown to negatively regulate PTEN protein levels in cancer cells [15]. Hence, we hypothesized that the DJ-1-PTEN-Akt-FoxO1 signaling cascade might regulate Ucp1 expression in BAT. We found that PTEN protein levels were significantly increased in BAT from DJ-1 KO mice (Figure 4a), whereas decreased in BAT from DJ-1 Tg mice (Figure 4a). Accordingly, Akt-mediated phosphorylation levels of FoxO1 decreased in DJ-1 KO BAT and increased in DJ-1 Tg BAT (Figure 4a). Further, we found Ucp1 transcription was markedly increased by DJ-1 knockdown and decreased by DJ-1 overexpression (Figure 4b). Chromatin immunoprecipitation (ChIP) *in vivo* analysis showed that FoxO1 is enriched on the Ucp1 promoter, which is increased in BAT of DJ-1 KO mice but decreased in BAT of DJ-1 Tg mice (Figure 4c). These results indicated that DJ-1 regulates Ucp1 expression via Akt signaling in BAT.

### DJ-1 promotes PTEN degradation through Mib2

It has been showed that DJ-1 negatively regulates PTEN [15], but the molecular mechanism is largely unknown. We next investigated how DJ-1 participated in the regulation of PTEN. There was no significant difference of PTEN mRNA levels between DJ-1 Tg or DJ-1 KO mice and WT mice (Figure 4d). Interestingly, we found that DJ-1 interacts with PTEN *in vitro* and *in vivo* (Figure 4e and f). Protein half-life analysis showed that overexpression of DJ-1 reduced PTEN stability (Figure 4g). Previous reports have shown that PTEN can be degraded via the proteasomal pathway [29, 30]. Here we also found that PTEN ubiquitination is increased by DJ-1 overexpression (Figure 4h), although as reduced by DJ-1 knockdown in brown adipose cells (Figure 4i).

**Figure 5:**
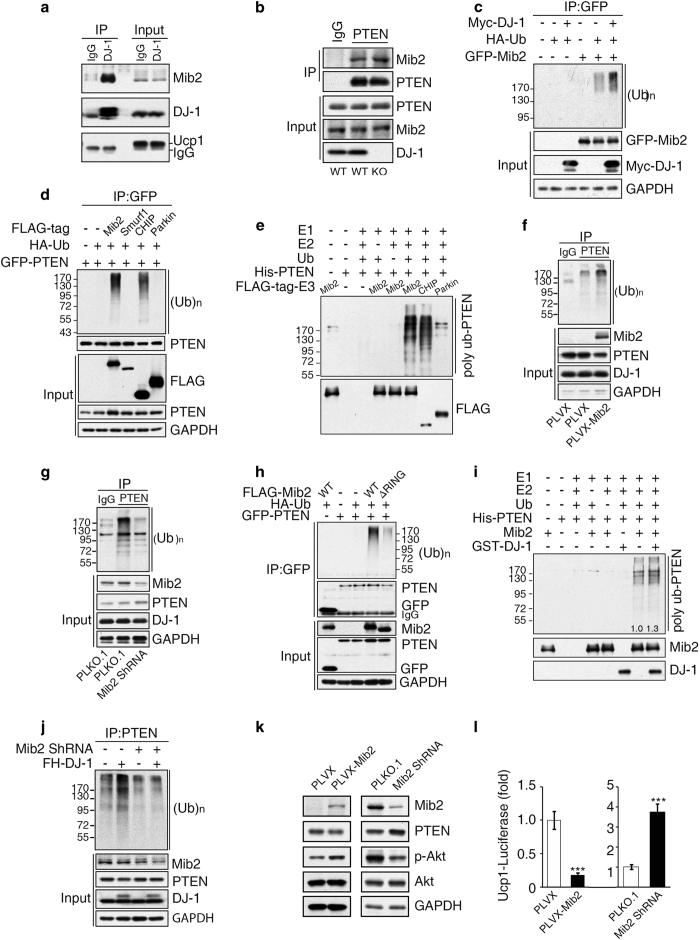
DJ-1 promotes PTEN degradation through E3 ligase Mib2. (**a**) Lysates of BAT were immunoprecipitated with DJ-1 antibody followed by immunoblotting with Mib2 antibody. (**b**) Endogenous co-IP assay of Mib2 and PTEN was performed in wild-type or DJ-1 KO brown adipocytes. (**c**) The ubiquitination of Mib2 was examined in HEK293T cells after transfected with GFP-tagged Mib2, HA-tagged ubiquitin and Myc-tagged DJ-1 or their control vectors. After 16 h transfected, the cells were treated with MG132 for 8 h. Cell lysates were immunoprecipitated with GFP antibody and immunoblotted with HA antibody. (**d**) The ubiquitination of PTEN was examined in HEK293T cells after transfected with the indicated plasmids. Cells were treated and analysis as in **c**. (**e**) Mib2 ubiquitination PTEN *in vitro*. His-PTEN was incubated with ubiquitin, E1, E2 (Ubc4) and ubiquitin ligase (Mib2, CHIP, Parkin), as indicated, and then it was analyzed by immunoblot using anti-His antibody. (**f**, **g**) Endogenous PTEN ubiqutination was examined in brown adipocytes with Mib2 overexpression (**f**) or Mib2 knockdown (**g**). Cells were treated with MG132 for 8 h. Cell lysates were immunoprecipitated with PTEN antibody and immunoblotted with ubiquitin antibody. (**h**) HEK293T cells were transfected with Flag-tagged WT Mib2 or RING domain truncation (ΔRING) Mib2 plasmids alone or together with HA-tagged ubiquitin, GFP-tagged PTEN. The cells then treated and analysis as in (**c**). (**i**) DJ-1 promotes Mib2 ubiquitination PTEN *in vitro*. His-PTEN was incubated with ubiquitin, E1, E2 (Ubc4), GST-DJ-1 and ubiquitin ligase (Mib2, CHIP and Parkin), as indicated, and then it was analyzed by immunoblot using anti-His antibody. GST-DJ-1 increased PTEN ubiqutination to 1.3-fold as indicated in the bottom of the panel. (**j**) Endogenous PTEN ubiqutination was examined in brown adipocytes with DJ-1-overexpression and/or Mib2 knockdown. Cells treated and analysis as in **f**. (**k**) Lysates of brown adipocytes with Mib2 overexpression or knockdown were immunoblotted with the indicated antibodies. (**l**) Ucp1-luciferase activity was analyzed in brown adipocytes with Mib2 overexpression or knockdown.

It was demonstrated that DJ-1 formed complex with PINK1/Parkin and promotes unfolded protein degradation [17]. To further explore the mechanism underlying DJ-1-mediated PTEN ubiquitination in BAT, we screened the potential E3 ligases that interact with DJ-1 and identified two E3 ligase, mind bomb-2 (Mib2) and smurf1 (Supplementary Figure S6A). We confirmed the interaction of DJ-1 and Mib2 in BAT and cells (Figure 5a, Supplementary Figure S6B and C) and interestingly, Mib2 also interacted with PTEN (Figure 5b and Supplementary Figure S6D). We also observed both DJ-1 and PTEN interacted with ankyrin repeat domain of Mib2 (Supplementary Figure S6E–G). Interestingly, DJ-1 could significantly promote auto-ubiquitination of Mib2 (Figure 5c). Next we found that Mib2, not Smurf1, was a specific E3 ligase for PTEN ubiquitination (Figure 5d and e). Moreover, PTEN ubiquitination is significantly increased by Mib2 overexpression and reduced by Mib2 knockdown in brown adipocytes (Figure 5f and g). Furthermore, Mib2 with RING domain deletion failed to promote PTEN ubiquitination (Figure 5h), indicating that Mib2-mediated PTEN ubiquitination depends on Mib2 E3 ligase activity. On the other hand, mutation of PTEN ubiquitination site K13 or K289 significantly blocked Mib2-mediated PTEN ubiquitination (Supplementary Figure S6H), indicating DJ-1 promoted PTEN ubiquitination through K13 and K289. In addition, DJ-1 could promote Mib2-mediated PTEN ubiquitination *in vitro* (Figure 5i). Importantly, DJ-1 overexpression-induced PTEN ubiquitination could be blocked by Mib2 knockdown (Figure 5j). Finally, we found that PTEN protein level and Ucp1 expression were decreased by Mib2 overexpression, whereas markedly increased by Mib2 knockdown in brown adipocytes (Figure 5k and l). Collectively, these results demonstrate that DJ-1 promotes PTEN degradation through Mib2 and DJ-1/Mib2/ PTEN-Akt-FoxO1 pathway regulates Ucp1 transcription in brown adipocytes.

### Ablation of Akt1 mitigates the obesity and BAT dysfunction induced by DJ-1 transgene

**Figure 6:**
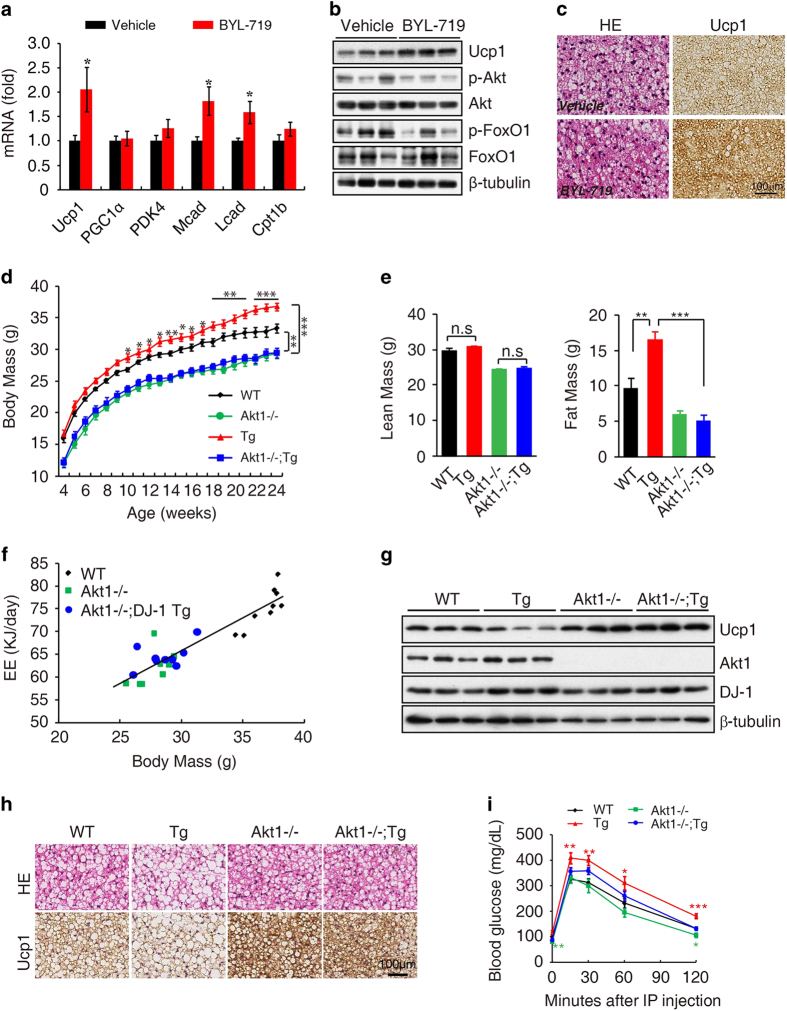
Ablation of Akt1 mitigates the obesity and BAT dysfunction induced by DJ-1 transgene. (**a**, **b**) The effect of BYL-719 on gene and protein expression in BAT. (**c**) HE or anti-Ucp1 antibody staining in BAT was performed after BYL-719 treatment. (**d**) Body mass of wild-type mice (WT), DJ-1 transgenic mice (Tg, Line 17 C57BL/6J background), Akt1 KO mice (Akt1^−/−^), DJ-1 transgenic and Akt1 knockout mice (Akt1^−/−^; Tg) fed on a chow diet for 24 weeks (WT, *n*=15; Akt1^−/−^, *n*=9; Tg, *n*=14; Akt1^−/−^; Tg, *n*=17). (**e**) Body lean and fat mass of 12-month-old indicated genotype mice measured by MRI (WT, *n*=7; Akt1^−/−^, *n*=11; Tg, *n*=8; Akt1^−/−^; Tg, *n*=8). (**f**) Statistical analysis by General Linear Model (GLM) and ANCOVA analysis of the indicated genotype mice energy expenditure (EE) (WT, *n*=9; Akt1^−/−^, *n*=8; Akt1^−/−^; Tg, *n*=9). (**g**) Lysates of BAT from mice of the genotypes indicated immunoblotted with Ucp1, Akt1, DJ-1 or β-tubulin antibodies. (**h**) HE and Ucp1 staining of BAT from 4-week-old mice of the indicated genotypes. (**i**) GTT results for 9-month-old indicated genotype mice fed on a chow diet (WT, *n*=8; Tg, *n*=7; Akt1^−/−^, *n*=7; Akt1^−/−^; Tg, *n*=8).

To further confirm whether DJ-1 regulates Ucp1 transcription through PI3K-Akt signaling *in vivo*, we investigated the effect of PI3K on Ucp1 expression and we found that four different PI3K inhibitors, including LY294002, GDC-0941, PI103, BYL-719 could increase the Ucp1 transcription in brown adipocytes (Supplementary Figure S7A). Moreover, oral administration of BYL-719, a PI3Kα inhibitor [31], resulted a significant upregulation of Ucp1 and the fatty acid oxidation-related genes in mice (Figure 6a–c). These results suggest that inhibition of PI3K enhances BAT activity.

There are three isoforms of Akt. Akt3 KO mice has overt developmental defect in brain [32]. Knockout of Akt2 led to insulin resistance and diabetes mellitus [33, 34]. Akt2 is a dominant isoform in adipocytes, but we found no obvious alteration in body mass and energy expenditure in Akt2 KO mice (Supplementary Figure S7B and C). Accordingly, Akt2 deletion has no effect on Ucp1 expression (Supplementary Figure S7D and E). Consistent with previous study [35, 36], we found that there was a significant reduction in body mass and fat mass in Akt1 KO mice compared with their WT littermates (Figure 6d and e and Supplementary Figure S7F). We next asked whether Akt1 contributes to DJ-1-mediated obesity and we found that Akt1 deletion significantly decreased DJ-1 transgene-induced obesity (Figure 6d and e and Supplementary Figure S7F).

**Figure 7:**
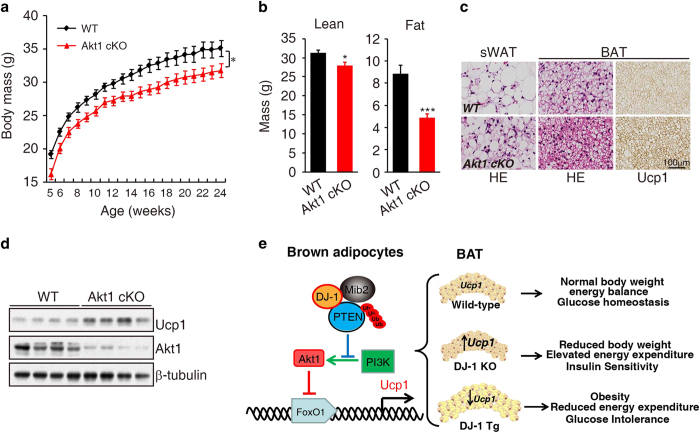
Specific knockout Akt1 in BAT increases Ucp1 expression. (**a**) Body mass of wild-type mice (WT) and Akt1 conditional knockout mice (Akt1 cKO) fed on a chow diet for 24 weeks(WT, *n*=17; Akt1 cKO, *n*=16). (**b**) MRI analysis body lean and fat mass from 25-week-old Akt1 BAT-specific knockout (Akt1 cKO) and WT littermates mice (WT, *n*=9; Akt1 cKO, *n*=9). (**c**) HE and Ucp1 staining of sWAT and BAT from 4-week-old mice of the Akt1 cKO and WT mice. (**d**) Lysates of BAT from 4-week-old WT and Akt1 cKO mice were subjected to immunoblotting with indicated antibody. (**e**) Model depicting the role of the DJ-1-Mib2-PTEN-Akt1- FoxO1 signaling cascade in energy balance and glucose homeostasis of BAT.

There is no change of energy expenditure between Akt1 KO and WT mice by General Linear Model (GLM) and ANCOVA analysis, which might be due to the analysis is not so sensitive in the mass of the WT group don't overlap circumstance (Figure 6f). However, Akt1 KO display increased Ucp1 expression (Figure 6g and h). Moreover, Akt1 KO restored the energy expenditure of DJ-1 Tg mice (Figure 6f), although no effects on food intake (Supplementary Figure S7G). Furthermore, we found that Akt1 KO reversed DJ-1 transgene-induced reduction of Ucp1 levels (Figure 6g and h). Accordingly, Akt1 deletion improved glucose tolerance (Figure 6i) but not insulin tolerance tests (Supplementary Figure S7H). To further clarify tissue-specific function of Akt1 in BAT, we generated BAT-specific KO mice by crossing conditional Akt1 KO (cKO) with *Myf5*-cre mice [25, 37]. We observed that Akt1 deficiency in BAT significantly reduced body mass and adiposity (Figure 7a–c) and increased Ucp1 expression in BAT (Figure 7c and d). In addition, Akt1 BAT-specific deletion improved glucose tolerance (Supplementary Figure S7I) but not insulin tolerance tests (Supplementary Figure S7J).

Taken together, we demonstrated that DJ-1-Mib2-PTEN-Akt-FoxO1 signaling pathway regulates Ucp1 transcription and the functional integrity of BAT, which is critical for energy balance and glucose homeostasis (working model in Figure 7e).

## Discussion

In this study, we show that DJ-1 maintains energy and glucose homeostasis by modulating the function of BAT and elucidate its molecular mechanism: (i) results from both gain-of-function (DJ-1 transgene) and loss-of-function (DJ-1 knockout) experiments demonstrate that DJ-1 regulates energy and glucose metabolism through modulating BAT function. (ii) BAT transplantation experiments further confirmed the tissue-specific function of DJ-1 in regulating BAT activity. (iii) Mechanistically, we found that DJ-1/Mib2 promote PTEN degradation, which in turn activates Akt and inhibits FoxO1-dependent transcription of Ucp1 in BAT.

DJ-1 was first identified as an oncogene [15]. Subsequent studies explored the multifaceted functions of DJ-1 in reproduction [8–10] and Parkinson’s diseases [16] as well as tumorigenesis [15]. Recently, DJ-1 has been linked to metabolism [38–41]. However, there have not been any previous systematic and comprehensive studies on the function and molecular mechanism of DJ-1 in energy and glucose homeostasis. In particular, the role of DJ-1 in BAT function has remained a mystery. BAT, which has a major role in energy consumption and thermogenesis, can be activated through the sympathetic nervous system upon cold exposure or nutritional excess treatment, increasing Ucp1 expression and energy expenditure [42]. In this report, we have systematically investigated the role and mechanism of DJ-1 in metabolism, especially in maintaining BAT function.

We found that DJ-1 deficiency increased energy expenditure and improved insulin sensitivity whereas transgenic expression of DJ-1 induced reduced energy consumption and glucose intolerance by alteration BAT activity. To our knowledge, this is the first study indicating that DJ-1 regulates BAT function and metabolic homeostasis. Contrast with our findings, recently, Jain D *et al.* [38] reports DJ-1 deletion induced glucose intolerance due to reduced islet beta cell and levels of insulin in 12–13 weeks old or 8 weeks old plus 2 weeks of HFD, In our study, glucose tolerance tests were performed from 3 months to 12 months old age, and we found that DJ-1 KO exhibits age-dependent glucose tolerance, although as DJ-1 Tg mice developed the age-dependent glucose intolerance due to alterations in BAT activity. It is likely that the improved insulin sensitivity was secondary to increased energy expenditure. Therefore, we proposed that the age is critical factor for maintaining glucose homeostasis in these mice.

During our manuscript was preparation, Shi SY *et al.* [41] reported that DJ-1 deletion modulates muscle ROS levels, thereby promoting mitochondrial uncoupling by increased Ucp3 expression. However, we found that the levels of Ucp3 expression had no significant changes in skeletal muscle under chow diet condition (data not shown). This difference may be explained by the fact that, in the present study, we analyzed skeletal Ucp3 expression at age of 12 months with chow diet condition, although Shi *et al.* analyzed at young age maximum at 5 month old with HFD condition. It has been shown that high-fat diet could affect the expression of uncoupling proteins expression [43]. DJ-1 modulates HFD-induced ROS production particularly in the skeletal muscle then Ucp3 can be activated by ROS [41, 44]. Therefore, it is not surprising that Ucp3 expression has no change under chow diet, although is increased in HFD in DJ-1 KO mice. In addition, Shi SY *et al.* did not observe a significant difference on Ucp1 expression in BAT between genotypes by using female mice fed with 3-month HFD treatment. However, we did observe the significant changes in Ucp1 expression in BAT at 12-month-old male mice at chow diet condition (Figure 3a–c and Figure 4a). The difference might be due to the difference in age and/or diet condition. Here, we argue that the expression of Ucp1 in BAT is regulated by DJ-1 in physiological condition, although the expression of Ucp3 in muscle is affected by pathological stimulation, including HFD. Interestingly, both studies reached a similar conclusion that DJ-1 deficiency improves glucose tolerance and DJ-1 maintains glucose homeostasis through regulating the whole body energy metabolism in different tissues under physiological and/or pathophysiological condition.

Extensive studies have shown that BAT functions as a thermogenic tissue by increasing energy expenditure and thus may be a therapeutic avenue for the treatment of obesity [1]. BAT transplantation has recently been shown to improve whole body metabolism, especially the maintenance of glucose homeostasis [5–7]. In our BAT transplantation experiments, we found that transplantation of DJ-1 KO BAT significantly ameliorated HFD-induced obesity, indicating that DJ-1 protein is detrimental for BAT activity. Furthermore, transplantation of DJ-1 Tg BAT failed to improve the functional integrity of endogenous BAT, confirming the function of DJ-1 in metabolic regulation. These results indicate that DJ-1 specifically modulates BAT activity to maintain energy and glucose homeostasis.

Ucp1, a gene selectively expressed in BAT, is localized in the inner mitochondrial membrane and is responsible for thermogenesis [26]. Ucp1 transcription is regulated by norepinephrine (NE), which is released by the sympathetic nervous system, and NE increases CREB/CEBPα complex-mediated Ucp1 expression in BAT [45]. In addition, other factors including Prdm16/ CEBPα [46], ERα [47], RIP40 [48] and FOXC2 [49] have also been reported to be involved in the regulation of Ucp1 expression. Recently, PTEN has been linked to the regulation BAT function [23, 25]. Here, we found that DJ-1 inhibits Ucp1 transcription and BAT activity *in vitro* and *in vivo* by negatively regulating PTEN signaling.

It has been reported that DJ-1 cooperates with Parkin and PINK1 to form the ubiquitin E3 ligase complex, promoting protein degradation [17]. Interestingly, Parkin and PINK1 both participate in the regulation of lipid metabolism [18, 19]. Recently, it has been reported PTEN ubiquitination was mediated by E3 ligases, including Nedd4a [30], WWP2 [50], XIAP [51] and CHIP [52]. In our hands, none of the above reported E3 ligases was responsible for DJ-1-mediated PTEN protein degradation (data not shown). In the present work, we identified Mib2 as a new E3 ligase for PTEN degradation, which is involved in DJ-1-mediated PTEN degradation in BAT and regulation of Ucp1 transcription. Mib2 is an E3 ligase in Notch [53] and NF-κB signaling [54]. Here we demonstrated that Mib2 regulates PI3K/Akt signaling through promoting PTEN degradation.

DJ-1 is reported to antagonize PTEN and mediate Akt activation in tumorigensis [15] and we found a conserved signaling cascade in the regulation of BAT function. We further found that Akt1 deletion increases Ucp1 expression in BAT. Interestingly, deletion of Akt1 in DJ-1 transgenic mice can rescue DJ-1 overexpression-induced Ucp1 inhibition and obesity. Hence, we have demonstrated that the DJ-1/Mib2/PTEN/ PI3K/Akt/FoxO1 signaling pathway regulates Ucp1 transcription in BAT.

Insulin signaling promotes glucose tolerance through increasing Akt phosphorylation and increasing GLUT4 translocation [55]. In this study, improved glucose homeostasis, increased PTEN protein stability and decreased Akt phosphorylation were observed in DJ-1 KO mice. In contrast, we observed opposing phenotype in DJ-1 Tg mice. As a putative phosphatase, PTEN plays a dominant inhibitory role for PI3K/Akt signaling. Conditional deletion PTEN in mice has been shown to affect liver, muscle, adipose tissue and pancreatic beta cells to induced insulin hypersensitivity [56–59] and PTEN mutations cause constitutive insulin sensitivity in human [60]. These phenotypes mechanistically coincide with Akt2, but not Akt1 knockout mice. However, PTEN transgenic mice showed reduced body weight, elevated energy expenditure and increased insulin sensitivity [23]. In addition, Akt1 knockout mice have improved insulin sensitivity [36, 61], which is similar as that of PTEN transgenic mice. These findings indicate that isoforms of Akt or tissue specificity of PTEN proteins are critical for the regulation of glucose metabolism. For example, we observed that deletion of Akt1, but not Akt2, significantly blocked DJ-1 transgene-induced glucose intolerance and obesity. Moreover, deletion of Akt1 with myf5-cre induced hyperactive BAT (Figure 7a–d). Interestingly, Lynes *et al.* reported specific ablation of the insulin receptor in the Myf5 lineage inhibited the differentiation of brown adipose progenitor cells. Myf5-cre-mediated insulin receptor deletion reduced BAT mass and UCP1 expression in BAT [62]. In addition, fat-specific knockout of the insulin receptor by the aP2-cre, which is mainly expressed in mature brown and white adipocytes [63], leads to the reduced white adipose tissue mass but no significant change in brown adipose tissue mass [64, 65]. The inconsistent phenotypes of insulin receptor in different cre-mice indicate that insulin receptor signaling could affect BAT mass mainly through regulating BAT progenitor differentiation. Here we found that Myf5-cre-mediated Akt1 deletion promoted UCP1 expression in BAT. The different phenotypes between insulin receptor deletion and Akt1 knockout might be due to that Akt1 regulates UCP1 expression only in mature brown adipocytes, whereas insulin receptor affects the brown adipose progenitor cells. These findings indicate that DJ-1-mediated metabolic regulation is Akt1-dependent in mature BAT.

In summary, the present work contributes to our understanding of the complicated regulatory network of metabolism. Our exogenous BAT transplantation data indicate that manipulating DJ-1 levels in BAT could be a possible therapeutic avenue for the treatment of obesity and diabetes.

## Materials and Methods

### Mice

DJ-1 knockout mice with a C57BL/6/CBA background were a kind gift from Dr Jie Shen of Harvard Medical School. Mice used here were backcrossed with C57BL/6 mice at least five times. A Flag-tagged human DJ-1 open reading frame was used to generate DJ-1 transgenic mice in a CD-1 background. The line 17 was maintained in the C57BL/6 background by backcrossing at least eight times. Akt1 knockout 129/Sv/C57BL/6 background mice were backcrossed with C57BL/6J mice more than five times. Myf5 cre mice were a kind gift from Dr Dahai Zhu. Myf5 cre mice, Akt1^flox/folx^ mice and Akt2 knockout mice were maintained in C57BL/6J background. Littermate controls were used in all experiments, and animals were random allocated to experimental groups and processed.

Mice were housed at 22–24 °C in a 12 h/12 h light–dark cycle with free access to water and were fed either with a standard chow diet or, when indicated, with a high-fat diet (45% of total calories from fat).

Mice were maintained in the Animal Care Facility at the Institute of Biophysics, Chinese Academy of Sciences, Beijing, and all experiments involving animals were approved by the Institutional Animal Care and Use Committee.

### Energy intake, energy expenditure and activity measurement

Mice were housed one animal per cage, and were given free access to food and water. Food intake and oxygen consumption were measured for two consecutive days using a TSE lab master system. Briefly, mice were placed in the measurement cages 4 h prior to data recording. Room temperature was maintained at 23 °C and light/dark cycles were of 12 h. The volume of O_2_ consumed (VO_2_) and CO_2_ eliminated (VCO_2_) was recorded every 12 min (in six simultaneous metabolic chambers, with a sample period of 6 min per cage, plus 1 min purge per cage). The respiratory quotient (RQ) was calculated as: RQ=VCO_2_/VO_2_. Energy expenditure (EE) was calculated as: EE=(3.815+(1.232×RQ))×VO_2_×1.44×24/1000). Ambulatory activity of each mouse was measured using the optical beam technique (Opto-M3; Columbus Instruments, Columbus, OH, USA) over a period of two days and is expressed as 24-hour average activity.

### Brown adipose tissue transplantation

After cervical dislocation of 5-week-old WT or DJ-1 KO donor mice, BAT was removed, peripheral white fat being excluded, and placed in sterile PBS. 0.2 g donor BAT was then transplanted as quickly as possible into the subcutaneous dorsal region, adjacent to the endogenous intra-scapular fat pad, of recipient mice. Recipient mice were anesthetized by intraperitoneal injection with avertin (400 mg kg^−1^ body weight). After transplantation, recipient mice and sham operation mice were given 200 μl 1 U ml^−1^ penicillin by intramuscular injection for seven consecutive days. After 1 week, recipient mice were fed on a high-fat diet for 16 weeks. The BAT from WT or DJ-1 Tg mice was transplanted to DJ-1 Tg mice and followed with similar procedure as the above except chow diet.

### Glucolse tolerance tests and Insulin tolerance tests

For glucose tolerance tests (GTT), animals were fasted for 16 h (1700–0900 hours) with free access to drinking water. The glucose level was assessed following intraperitoneal glucose injection (2.0 g kg^−1^). Serum glucose levels were determined immediately before and 15, 30, 60 and 120 min after glucose injection using a glucometer (OneTouch Ultra, Bayer, Berlin, Germany). For insulin tolerance tests (ITT), mice were fasted for 4 h (9:00–13:00) and then intraperitoneally injected with human insulin (0.75 U kg^−1^ for C57BL/6J background mice and 1.5 U kg^−1^ for CD-1 background mice). Blood glucose levels were determined immediately before and 15, 30, 60 and 120 min after insulin injection.

### Histological analysis

Brown adipose tissue, white adipose tissue and livers were fixed overnight in 4% paraformaldehyde, embedded in paraffin blocks and sectioned. Tissue sections were stained with hematoxilin/eosin or with anti-Ucp1 (ab10983, Abcam, Cambridge, UK) following standard procedures.

### PI3K inhibitors treatment

For *in vitro* assays, 10 μm LY294002, GDC0941, BYL-719 and 1 μm PI103 were used to treat brown adipocytes precursor cells. All PI3K inhibitor were obtained from Selleck (Houston, TX, USA), and dissolved in DMSO. For *in vivo* experiments, BYL-719 dissolved in *N*-methyl pyrrolidone, polyethylene glycol 300, Kolliphor HS15, and water (10%:30%:20%:40%, v/v) and was orally administrated to two months old C57BL/6J male mice at a dose of 50 mg kg^−1^. Mice were anaesthetized and killed 8 h later and tissues were extracted and analyzed.

### Isolation, culture, immortalization and differentiation of brown adipocyte precursors

Brown adipocyte precursors were isolated from newborn C57BL/6J mice by collagenase digestion and cultured in high glucose DMEM medium (20% FBS, 20 mm HEPES, 1× penicillin/streptomycin). The cells were immortalized by infection with a retroviral vector encoding SV40 Large T antigen. Primary brown adipocyte precursors from newborn wild-type and DJ-1 transgenic mice were differentiated by growing in culture medium supplemented with 20 nM insulin and 1 nm T3 (differentiation medium). After reaching confluence, cells were cultured for 48 h in differentiation medium further supplemented with 0.5 mm isobutylmethylxanthine, 0.5 mm dexamethasone and 0.125 mm indomethacin. After culturing for 4 more days in differentiation medium, cells exhibited a fully differentiated phenotype with massive accumulation of multilocular fat droplets.

### Quantitative RT-PCR

Total RNA from tissues or cells was extracted using TRIZOL (Invitrogen, Waltham, MA, USA). Reverse transcription was performed using random primers. Quantitative real time-PCR was performed using TransStart Green qPCR SuperMix (Transgen Biotecth, Beijing, China) in a Stratagene Mx3005P (Agilent Technologies, Santa Clara, CA ,USA). Primer sequences are given below. The housekeeping gene used for input normalization of all the qPCR data was β-actin. mRNA expression was measured by quantitative PCR using the Delta-Delta CT method. Primers for quantitative PCR: β-actin-Fw: 5′- GGCTGTATTCCCCTCCATCG-3′; β-actin-Rv: 5′- CCAGTTGGTAACAATGCCATGT-3′; Ucp1-Fw: 5′- AGG CTTCCAGTACCATTGGT-3′; Ucp1-Rv: 5′- CTGAGTGAGGCAAAGCTGATT T-3′; PGC1α-Fw: 5′- TATGGAGTGACATAGAGTGTGCT-3′; PGC1α-Rv: 5′- CCACTTCAATCCACCCAGAAAG-3′; PDK4-Fw: 5′- AGGGAGGTCGAGCTGTTCTC-3′; PDK4-Rv: 5′- GGAGTGTTCACTAAGCGGTCA-3′; Prdm16-Fw: 5′- CCACCAGCGAGGACTTCAC-3′; Prdm16-Rv: 5′- GGAGGACTCTCGTAGCTCGAA-3′; CPT1b-Fw: 5′- GCACACCAGGCAGTAGCTTT-3′; CPT1b-Rv: 5′- CAGGAGTTGATTCCAGACAGGTA-3′; Mcad-Fw: 5′- AGGGTTTAGTTTTGAGTTGACGG-3′; Mcad-Rv: 5′- CCCCGCTTTTGTCATATTCCG-3′; Lcad-Fw: 5′- TCTTTTCCTCGGAGCATGACA-3′; Lcad-Rv: 5′- GACCTCTCTACTCACTTCTCCAG-3′; Cebpα-Fw: 5′- CAAGAACAGCAACGAGTACCG-3′; Cebpα-Rv: 5′- GTCACTGGTCAACTCCAGCAC-3′; Cebpβ-Fw: 5′- CAAGCTGAGCGACGAGTACA-3′; Cebpβ-Rv: 5′- AGCTGCTCCACCTTCTTCTG-3′; DJ-1-Fw: 5′- GCTTCCAAAA GAGCTCTGGTC-3′; DJ-1-Rv: 5′- ACATCACGGCTACACTG-3′; PTEN-Fw: 5′- TGGATTCGACTTAGACTTGACCT-3′; PTEN-Rv: 5′- GCGGTGTCATAATGTCTCTCAG-3′; TH-Fw: 5′-CCAAGGTTCATTGGACGGC-3′; TH-Rv: 5′-CTCTCCTCGAATACCACAGCC-3′; Agrp-Fw: 5′-ATGCTGACTGCAATGTTGCTG-3′; Agrp-Rv: 5′-CAGACTTAGACCTGGGAACTCT-3′; Npy-Fw: 5′-ATGCTAGGTAACAAGCGAATGG-3′; Npy-Rv: 5′-TGTCGCAGAGCGGAGTAGTAT-3′; Pomc-Fw: 5′-ATGCCGAGATTCTGCTACAGT-3′; Pomc-Rv: 5′-CCACACATCTATGGAGGTCTGAA -3′; Stat3-Fw: 5′-CAATACCATTGACCTGCCGAT-3′; Stat3-Rv: 5′-GAGCGACTCAAACTGCCCT-3′; Leprb-Fw: 5′- TGGTCCCAGCAGCTATGGT-3′; Leprb-Rv: 5′- ACCCAGAGAAGTTAGCACTGT-3′.

### Plasmids construct and screen stable expression cell

DNA fragments corresponding to full-length or ΔRING (deletion of the RING domain) Mib2 (isoform2) were amplified from a mouse brown adipose precursors cDNA library by PCR and inserted into p3×FLAG-CMV-10 Expression Vector (Sigma-Aldrich, St Louis, MO, USA) using the NotI and EcoRI restriction sites. FLAG-HA tagged DJ-1(mouse) was inserted retroviral vector PQCXIH using the *Not*I and *BamH*I restriction sites. To generate DJ-1 overexpression cells, immortalized brown fat precursors were infected with the retroviral vector PQCXIH encoding FLAG-HA-DJ-1 (mouse) and selected with hygromycin. To generate DJ-1 stable knockdown cells, immortalized brown fat precursors were infected the lentiviral vector PLKO.1 encoding short hairpin RNA for mouse DJ-1. shRNA targeting sequences used were shRNA1#: 5′-ATCTGGGTGCACAGAATTTAT-3′; and shRNA2#: 5′-CCATACGATGTGGTGGTTCTT-3′. Mib2 stable knockdown cell used shRNA targeting sequences TCGAAGGATGAAGAAGTGTAT by the similar methods. To generate Mib2 stable overexpression cell, immortalized brown fat precursors were infected with the lentiviral vector PLVX-AcGFP-N1 encoding FLAG-Mib2 (mouse) and selected with puromycin.

### Co-immunoprecipitation

Cells for co-immunoprecipitation were lysed in IP buffer containing 50 mm Hepes, pH 7.9, 150 mm NaCl, 10% Glycerol, 1% Triton-100, 1.5 mm MgCl_2_, 0.1 m NaF, 1 mm EGTA, 2 mm phenylmethylsulfonyl fluoride, 2 μg ml^−1^ aprotinin and leupeptin and 1 mm sodium vanadate. Lysates were centrifuged at 12 000 *g* for 15 min at 4 °C prior to immunoprecipitation and precleared with 2 μl IgG and protein G agarose beads at 4 °C for 2 h. Following the removal of the beads by centrifugation, lysates were incubated with appropriate antibodies in the presence of 30 μl of protein G agarose beads for at least 2 h 4 °C. The beads were washed with IP buffer four times and the immunoprecipitates were subjected to immunoblotting.

### Immunoblotting

Tissues or cells were for immunoblotting were lysed in buffer containing 50 mm Hepes, pH 7.4, 150 mm NaCl, 1% Nonidet P-40, 0.1% deoxycholate, 0.05% SDS, 0.1 m NaF, 1 mm EGTA, 2 mm phenylmethylsulfonyl fluoride, 2 μg ml^−1^ aprotinin and leupeptin and 1 mm sodium vanadate. Protein concentration was determined using a Bio-Rad protein assay kit (Bio-Rad, Hercules, California, USA). Proteins were separated on a 10% polyacrylamide gel and transferred to a methanol-activated PVDF membrane (GE Healthcare, Little Chalfont, UK). The membrane was blocked for one hour in TBST containing 5% milk and subsequently probed with primary antibodies overnight at 4 °C. After incubating for 1 h with goat-anti-mouse or goat-anti-rabbit HRP-conjugated secondary antibodies (GE Healthcare), protein level was detected with Super Signal West Pico and Femto Luminol reagents (Thermo Scientific, Waltham, MA, USA). The antibodies used were anti-phosphor-Akt (Ser473; #4060, Cell Signaling, Cambridge, MA, USA), anti-Akt (#4691, Cell Signaling), anti-Akt1 (#2938, Cell Signaling), anti-FoxO1 (sc-11350, Santa Cruz, Dallas, TX, USA), anti-Akt2 (#3063, Cell Signaling), anti-phosphor-FoxO (Thr24-FoxO1/ Thr32-FoxO3a; #9464, Cell Signaling), anti-phosphor-HSL (Ser660; #4126, Cell Signaling), anti-HSL (#4107, Cell Signaling), anti-PTEN (#9188, Cell Signaling), anti-DJ-1 (#5933, Cell Signaling), anti-Ucp1 (ab10983), anti-Ubiquitin (#3936, Cell Signaling), anti-Mib2 (Ls-C295379, LifeSpan BioSciences, Seattle, WA, USA), anti-FLAG (Sigma), anti-GFP (Invitrogen), anti-HA (Santa Cruz), anti-β-tubulin (CW0098A, CWBiotech, Beijing, China), anti-β-actin (60008-1-Ig, Proteintech Group, Campbell Park, Chicago, IL, USA) and anti-GAPDH (CW0266A, CWBiotech).

### *In vivo* PTEN ubiquitylation assays

For PTEN ubiquitination assays, the cells were treated with 20 μg ml^−1^ of the proteasome inhibitor MG132 (Selleck) for 8 h. Then, the cells were lysed in buffer containing 50 mm Hepes, pH 7.4, 150 mm NaCl, 1% Nonidet P-40, 0.1% deoxycholate, 0.05% SDS, 0.1 M NaF, 1 mm EGTA, 2 mm phenylmethylsulfonyl fluoride, 2 μg ml^−1^ aprotinin and leupeptin, 1 mm sodium vanadate and 20 μg ml^−1^ MG132. The lysates were centrifuged and supernatant were incubate with PTEN or GFP antibody for 4 h and protein G beads for further 2 h. Then the beads were washed 4 times with lysis buffer, following by SDS-PAGE electrophoresis and immunoblotting.

### *In vitro* ubiquitination

The reaction was carried out at 37 °C for 1 h in 50 μl reaction buffer (20 mm Tris-HCl, pH 7.4, 1 mm DTT, 10 mm MgCl_2_, 1 mm ATP) containing the following components: 0.5 μg of ubiquitin, 250 ng E1, 500 ng of E2( Ubc4) and 0.5 μg of His-PTEN. FLAG-tagged E3 ligase were purification from HEK293T cells which transfected with FLAG-tagged Mib2, CHIP or Parkin. After ubiquitination reaction, the samples were directly boiled with SDS-PAGE loading buffer and analyzed by immunoblotting against anti-HA antibody (Santa Cruz). The samples were separated on a 8% SDS-PAGE followed by immunoblot analysis using His antibody (CW0286, CWBiotech).

### Luciferase reporter assay

The Ucp1-luciferase reporter gene was a kind gift from Dr Mark Christian of the University of Warwick. 0.5 μg Ucp1-luciferase reporter genes and 0.2 μg TK-Renilla were co-transfected into pre-brown adipocytes cells in a 24-well plate using lipofectamin-2000 (Invitrogen). After 24 h, cell extracts were obtained and firefly and Renilla luciferase activities were measured using a Promega Dual-Luciferase reporter system.

### Chromatin immunoprecipitation

Mice were perfused with normal salting following 37% formaldehyde after starvation 24 h at 8 °C. BAT was removed, peripheral white fat being excluded, and placed in 1 ml cell lysis buffer (5 mm Pipes (KOH), pH 8.0, 85 mm KCl, Nonidet P-40 (0.5% vol/vol), 1× protease inhibitors], tissue were cut into small pieces by scissors following homogenates by homogenizer. Removed supernatant and collected nuclear pellet by centrifugation and lysed in 300 μl nuclear lysis buffer (1% (w/v) SDS, 5 mm EDTA, 50 mm Tris·Cl, pH 8.0, 1× protease inhibitors). The mixture was then sonicated to produce soluble chromatin with an average size of 300–1500 bp. After centrifugation at 12 000×g for 20 min, the supernatant was collected. Soluble chromatin was diluted 10-fold in dilution buffer [1.11% Triton X-100, 1.67 mm EDTA, 167 mm NaCl, 16.7 mm Tris·Cl, pH 8.0, 1× protease inhibitor] and then precleared by incubating 1 ml diluted chromatin with salmon protein-A agarose beads (50 μl 50% slurry in 10 mm Tris·Cl, pH 8.1, 1 mm EDTA) for 2 h at 4 °C. The supernatant was collected by centrifugation, 20% of which was retained for use as the total input control. 4 μg ChIP-grade anti-FoxO1 (ab39670, Abcam) antibody and 2 μg anti-FoxO1 (sc-11350, Santa Cruz) antibody was added to the lystaes and incubated overnight at 4 °C with agitation. To collect the immunocomplex, 60 μl of salmon protein-A agarose beads were added to the samples and incubated for 4 h at 4 °C. The beads were collected and washed sequentially for 5 min each in low salt wash buffer [0.1% SDS, 1% Triton X-100, 2 mm EDTA, 20 mm Tris pH 8.0, 150 mm NaCl, 1×], high salt wash buffer [0.1% SDS, 1% Triton X-100, 2 mm EDTA, 20 mm Tris, pH 8.0, 500 mm NaCl, 3×), LiCl buffer (0.25 m LiCl, 1% Nonidet P-40, 1% deoxycholate, 10 mm Tris·Cl, 1 mm EDTA; 2×) and Tris-EDTA buffer (10 mm Tris pH 8.0, 1 mm EDTA, 2×). The bound protein-DNA immunocomplexes were eluted with 250 μl elution buffer (1% SDS, 0.1 m NaHCO_3_) and reversed formaldehyde cross-links by adding 1 μl 10 mg ml^−1^ RNase and 5 m NaCl to a final concentration of 0.3 m to eluants and incubating in a 65 °C water bath for 5 h. After adding 2.5 volumes of 100% ethanol and precipitating overnight at −20 °C, DNA was pelleted and resuspended in 100 μl of water. 2 μl of 0.5 m EDTA, 4 μl 1 m Tris, pH 6.5 and 1 μl of 20 mg ml^−1^ Proteinase K were added and incubated for 2 h at 55 °C. DNA was purified using a QIAquick PCR Purification Kit (Qiagen) and eluted in 50 μl TE buffer. Two microliter of the eluted DNA sample was used in qPCR reactions. The primers used for amplifications were as follows:

Ucp1 promoter (forward): 5′- CTGTTGTTGCTGCTGCTGTT-3′

Ucp1 promoter (reverse): 5′- GGAAGCTGCAAGACCTATGG-3′

### Statistical analysis

Comparisons between two groups were made by unpaired two-tailed Student’s *t*-tests. Data for body mass change over time were analyzed by an unbalanced two-way analysis of variance (ANOVA). All values are expressed as mean±s.e.m. *P<0.05, **P<0.01, ***P<0.001 for two-tailed Student’s *t*-tests, unless stated otherwise.
