# Supplementary material for: DJ-1 maintains energy and glucose homeostasis by regulating the function of brown adipose tissue
Source: Cell Discov. 2017 Feb 14;3:16054–. doi: 10.1038/celldisc.2016.54 (PMC5309696; doi:10.1038/celldisc.2016.54)
Supplement: Supplementary Information [file celldisc201654-s1.pdf]

Supplementary information

Supplementary Figure S1 Ablation DJ-1 reduced fat mass, increased energy expenditure and improved insulin sensitivity

A, Mass of indicated organ and fat tissue from 12-month-old WT and DJ-1 KO mice. **B, C,** Body and Tibia length of 12-month-old WT and DJ-1 KO mice（WT, n=5; KO, n=4）. **D,** The levels of free fatty acid in plasma from 10-12 months old mice. **E,** Respiratory quotient (VCO_2/VO_2) measured in 6 months old adult males (n=8 per genotype). **F,** Daily food intake and **G** locomotive activity of 6-month-old male littermates fed on a chow diet (n=6 per genotype). **H-K,** GTT results for WT and DJ-1 KO mice fed on a chow diet at the indicated age. **L-O,** ITT results for WT and DJ-1 KO mice fed on a chow diet at the indicated age.

Supplementary Figure S2 DJ-1 KO mice resists high fat diet induced obesity

A, Lean body and fat mass of HFD mice as measured by MRI (WT, n=7; KO, n=5). **B,** Mass of eWAT, sWAT and BAT from HFD mice. **C,** Histology of adipose tissue and liver. **D,** Respiratory quotient (VCO_2/VO_2) measured in 6 months old high fat diet induced adult males (WT, n=13; KO, n=10).

Supplementary Figure S3 the DJ-1 transgene induced obesity, decreased energy expenditure and glucose intolerance

A, Schematic of the *CMV*-FLAG-hDJ-1 transgene constructs. **B,** Immunoblotting of DJ-1 in major tissues or organ from DJ-1 transgenic mice (Tg) and age-matched littermate wild-type mice (WT). **C,** Mass of indicated organ and fat tissue from 10-month-old WT and DJ-1 transgenic mice. **D,** The level of free fatty acid in plasma from seven months old indicated genotype mice. **E,** Respiratory quotient (VCO_2/VO_2) measured in 8-month-old male mice. **F,** Daily food intake and **G** locomotive activity of 8-month-old male littermates fed on a chow diet (n=6 per genotype). **H,** GTT results for WT and DJ-1 Tg mice fed on a chow diet at the indicated age. **I,** ITT results for WT and DJ-1 Tg mice fed on a chow diet at the indicated age.

Supplementary Figure S4 DJ-1 regulates Ucp1 expression in brown adipocytes

A, Gene expression in hypothalamus from 10-12 month old WT and DJ-1 KO mice (n=5 per genotype). **B,** Gene expression in hypothalamus from seven months old WT and DJ-1 transgenic mice (n=5 per genotype). **C,** The mRNA level of tyrosine hydroxylase in BAT from indicated genotype mice (n=6 per genotype). **D,** Lysates of BAT and eWAT from 12 month-old indicated genotype mice were immunoblotted with the indicated antibodies. **E,** Primary brown fat precursors were isolated from DJ-1 KO and littermates WT mice (upper panel) and DJ-1 Tg and littermates WT mice (bottom panel) on the first day after birth and differentiated in vitro for 6 days. Cells were stained with oil red O and photographed. **F,** Gene expression in differentiated brown fat cells. **G,** Gene expression in differentiated brown fat cells.

Supplementary Figure S5 BAT transplantation resists obesity and improves glucose homeostasis

A, Mass of fat tissue and the liver in recipient mice (sham, WT BAT or DJ-1 KO BAT). **B,** WAT and liver histology in recipient mice (sham, WT BAT or DJ-1 KO BAT). **C,** ITT assays of 20-week-old and HFD-induced recipient mice (sham, WT BAT or DJ-1 KO BAT). **D,** Mass of eWAT, sWAT and BAT from recipient DJ-1 transgenic mice subcutaneously transplanted with BAT from WT or DJ-1 transgenic mice. **E,** WAT histology of mice in (**D**). **F,** ITT assay results for 26-week-old DJ-1 transgenic mice transplanted with BAT.

Supplementary Figure S6 Mib2 physically interacts with DJ-1 and PTEN

A, Lysates of brown adipocytes stably expressed with FLAG-HA tagged DJ-1 were immunoprecipitation with FLAG antibody and eluted with FLAG peptide, followed by LC-MS/MS analysis. The E3 ligases were listed. **B, D,** Co-IP assay of Mib2 and DJ-1 or PTEN was performed in cells transfected with the indicated plasmids. **C,** Endogenous co-IP assay of Mib2 and DJ-1 was performed in brown adipocytes. **E,** Schematic drawing of Mib2 protein with predicted domains and deletion mutants. **F,**

G, Mapping the interaction domains between Mib2 and DJ-1 or PTEN. Co-IP of GFP-DJ-1 or GFP-PTEN and FLAG-Mib2 from cells transfected with vector, FLAG-tagged WT Mib2 (Full length), or FLAG-tagged Mib2 deletion mutants containing amino acids 1-427, 428-797, or 798-921 of Mib2. **H,** The ubiquitination of of WT or mutant PTEN was examined in cells transfected with the indicated plasmids.

Supplementary Figure S7 PI3K inhibition and Akt1 contribution to Ucp1 expression and but not Akt2

A, The effect of PI3K inhibitor LY294002, GDC-0941, PI103 or BYL-719 or forskolin on Ucp1-luciferase reporter activity in immortalized brown adipose precursors. Right panel: cell lysates was immunoblotted with the indicated antibodies. **B,** Changes of body mass with age in Akt2 KO and WT mice. **C,** Statistical analysis by General Linear Model (GLM) and ANCOVA analysis of the 13 weeks old male indicated genotype mice energy expenditure (EE) (WT n=5, Akt2 KO n=7). **D,** Lysates of BAT from WT and Akt2 KO mice were subjected to immunoblotting with indicated antibody. **E,** HE or anti-Ucp1 antibody staining in BAT was performed in Akt2 KO and WT mice. **F,** Mass of eWAT, sWAT and BAT from 12-month-old indicated genotype mice (WT, n=11; Akt1-/-, n=10; Tg, n=10; Akt1-/-; Tg, n=16). **G,** Daily food intake of 8-month-old indicated genotype mice (WT, n=8; Akt1-/-, n=6; Tg, n=8; Akt1-/-; Tg, n=6). **H,** ITT results for 9-month-old WT, DJ-1 Tg, Akt1 KO , DJ-1 Tg and Akt1 KO mice fed on a chow diet (WT, n=8; Tg, n=7; Akt1-/-, n=7; Akt1-/-; Tg, n=8). **I,** GTT and **J,** ITT results for 24 weeks old WT and Akt1 cKO mice fed on a chow diet (WT, n=6; Akt1 cKO, n=7).

Figure S1

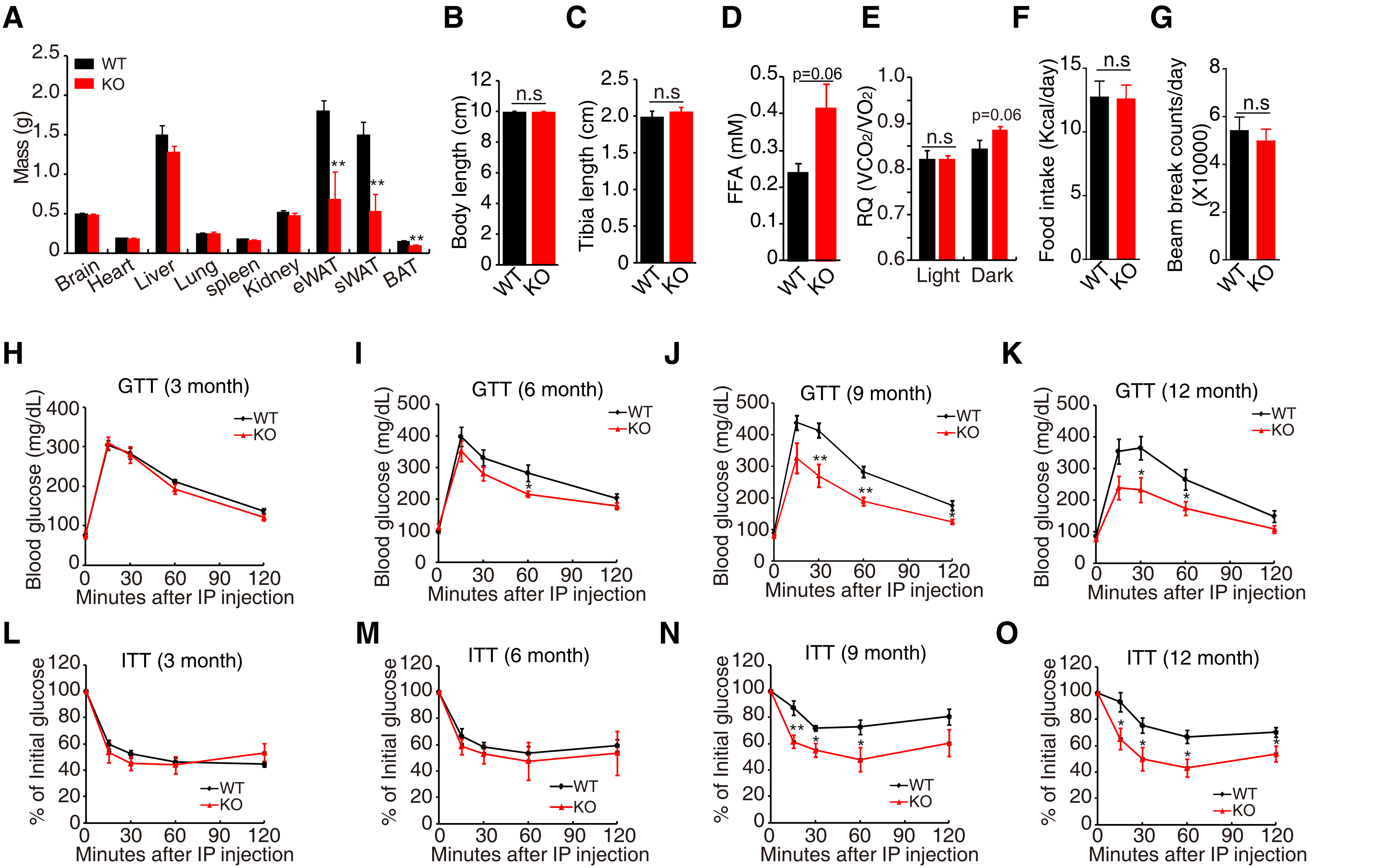
A
Mass (g)
WT
KO
Brain
Heart
Liver
Lung
spleen
Kidney
eWAT
sWAT
BAT
**
B
Body length (cm)
n.s
C
Tibia length (cm)
n.s
D
FFA (mM)
p=0.06
E
RQ (VCO2/VO2)
n.s
p=0.06
Light
Dark
F
Food intake (Kcal/day)
n.s
G
Beam break counts/day (X10000)
n.s
H
GTT (3 month)
I
GTT (6 month)
J
GTT (9 month)
K
GTT (12 month)
Blood glucose (mg/dL)
Minutes after IP injection
L
ITT (3 month)
M
ITT (6 month)
N
ITT (9 month)
O
ITT (12 month)
% of Initial glucose
Minutes after IP injection

Figure S2

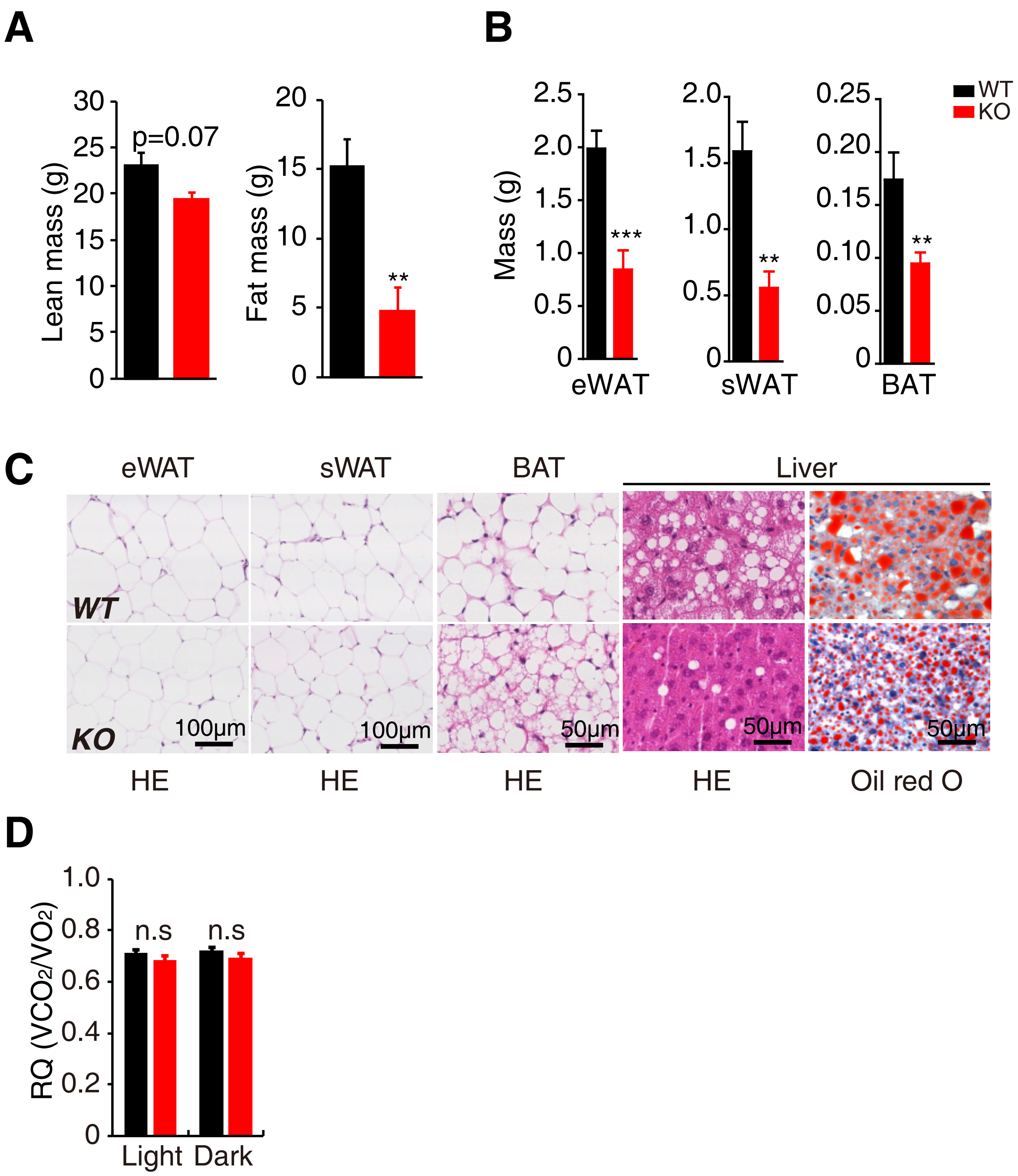
A
30
25
20
15
10
5
0
Lean mass (g)
p=0.07
20
15
10
5
0
Fat mass (g)
**
B
Mass (g)
2.5
2.0
1.5
1.0
0.5
0
***
eWAT
2.0
1.5
1.0
0.5
0
**
sWAT
0.25
0.20
0.15
0.10
0.05
0
**
BAT
WT
KO
C
eWAT
sWAT
BAT
Liver
WT
KO
100µm
100µm
50µm
50µm
50µm
HE
HE
HE
HE
Oil red O
D
1.0
0.8
0.6
0.4
0.2
0
RQ (VCO2/VO2)
n.s
n.s
Light
Dark

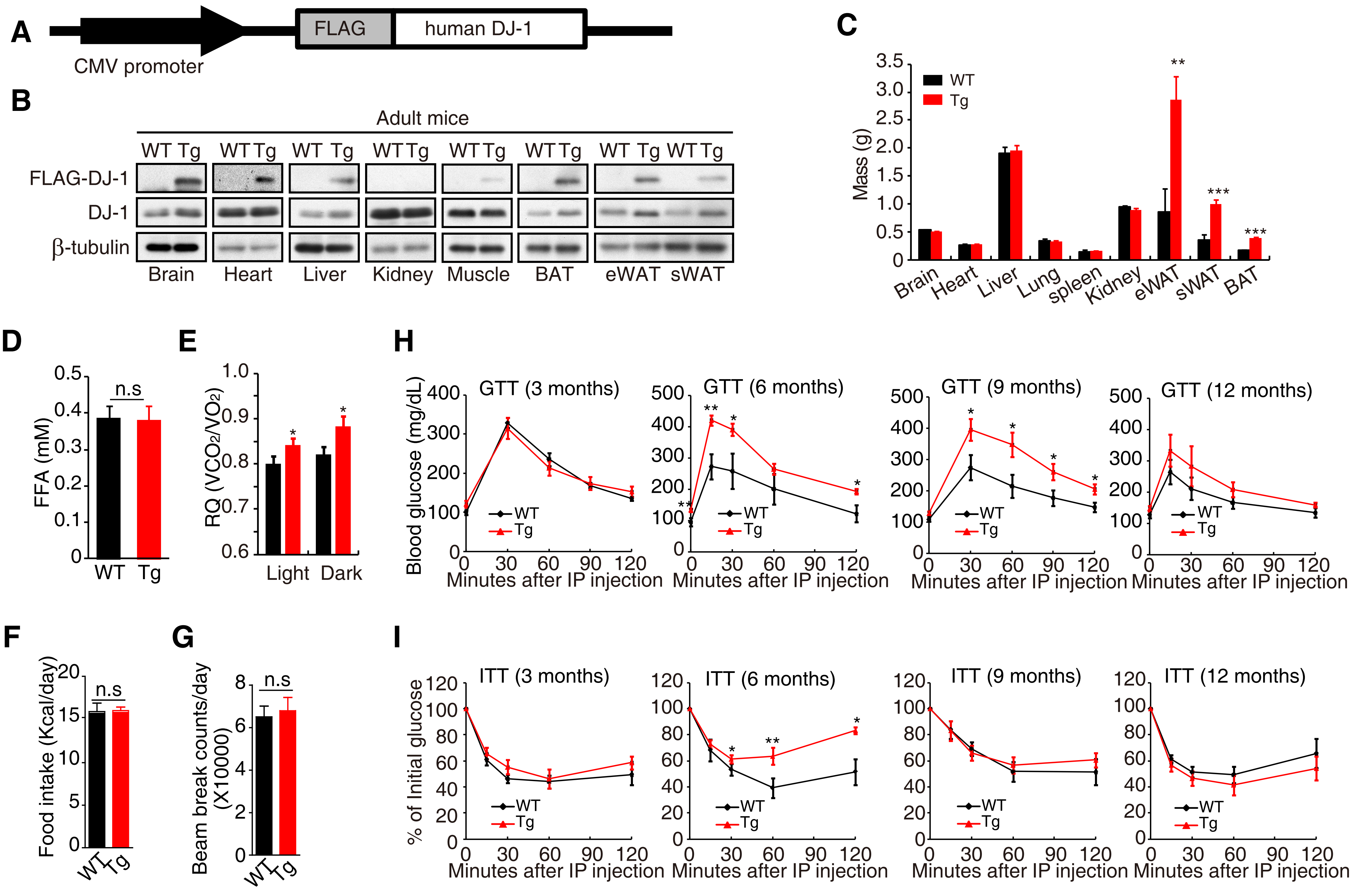
A
CMV promoter
FLAG
human DJ-1
B
Adult mice
WT Tg WT Tg WT Tg WT Tg WT Tg WT Tg WT Tg WT Tg
FLAG-DJ-1
DJ-1
β-tubulin
Brain Heart Liver Kidney Muscle BAT eWAT sWAT
C
Mass (g)
WT
Tg
Brain Heart Liver Lung spleen Kidney eWAT sWAT BAT
D
FFA (mM)
n.s
WT Tg
E
RQ (VCO2/VO2)
Light Dark
H
Blood glucose (mg/dL)
GTT (3 months)
GTT (6 months)
GTT (9 months)
GTT (12 months)
Minutes after IP injection
F
Food intake (Kcal/day)
n.s
G
Beam break counts/day (X10000)
n.s
I
% of Initial glucose
ITT (3 months)
ITT (6 months)
ITT (9 months)
ITT (12 months)
Minutes after IP injection

Figure S4

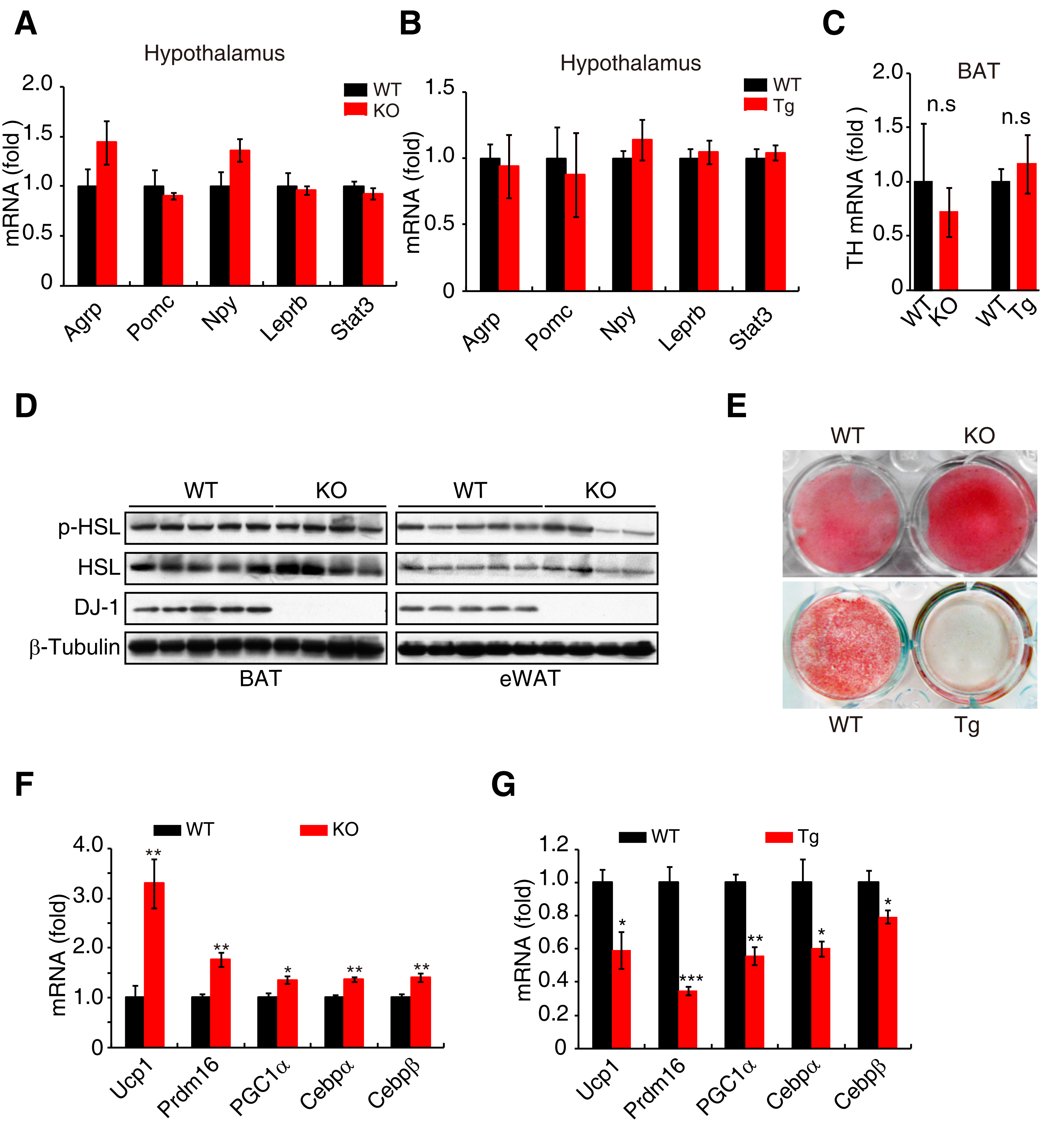
A
Hypothalamus
WT
KO
mRNA (fold)
2.0
1.5
1.0
0.5
0
Agrp
Pomc
Npy
Leprb
Stat3
B
Hypothalamus
WT
Tg
mRNA (fold)
1.5
1.0
0.5
0
Agrp
Pomc
Npy
Leprb
Stat3
C
BAT
n.s
n.s
TH mRNA (fold)
2.0
1.5
1.0
0.5
0
WT
KO
WT
Tg
D
WT
KO
WT
KO
p-HSL
HSL
DJ-1
β-Tubulin
BAT
eWAT
E
WT
KO
WT
Tg
F
WT
KO
mRNA (fold)
4.0
3.0
2.0
1.0
0
Ucp1
Prdm16
PGC1α
Cebpα
Cebpβ
G
WT
Tg
mRNA (fold)
1.2
1.0
0.8
0.6
0.4
0.2
0
Ucp1
Prdm16
PGC1α
Cebpα
Cebpβ

Figure S5

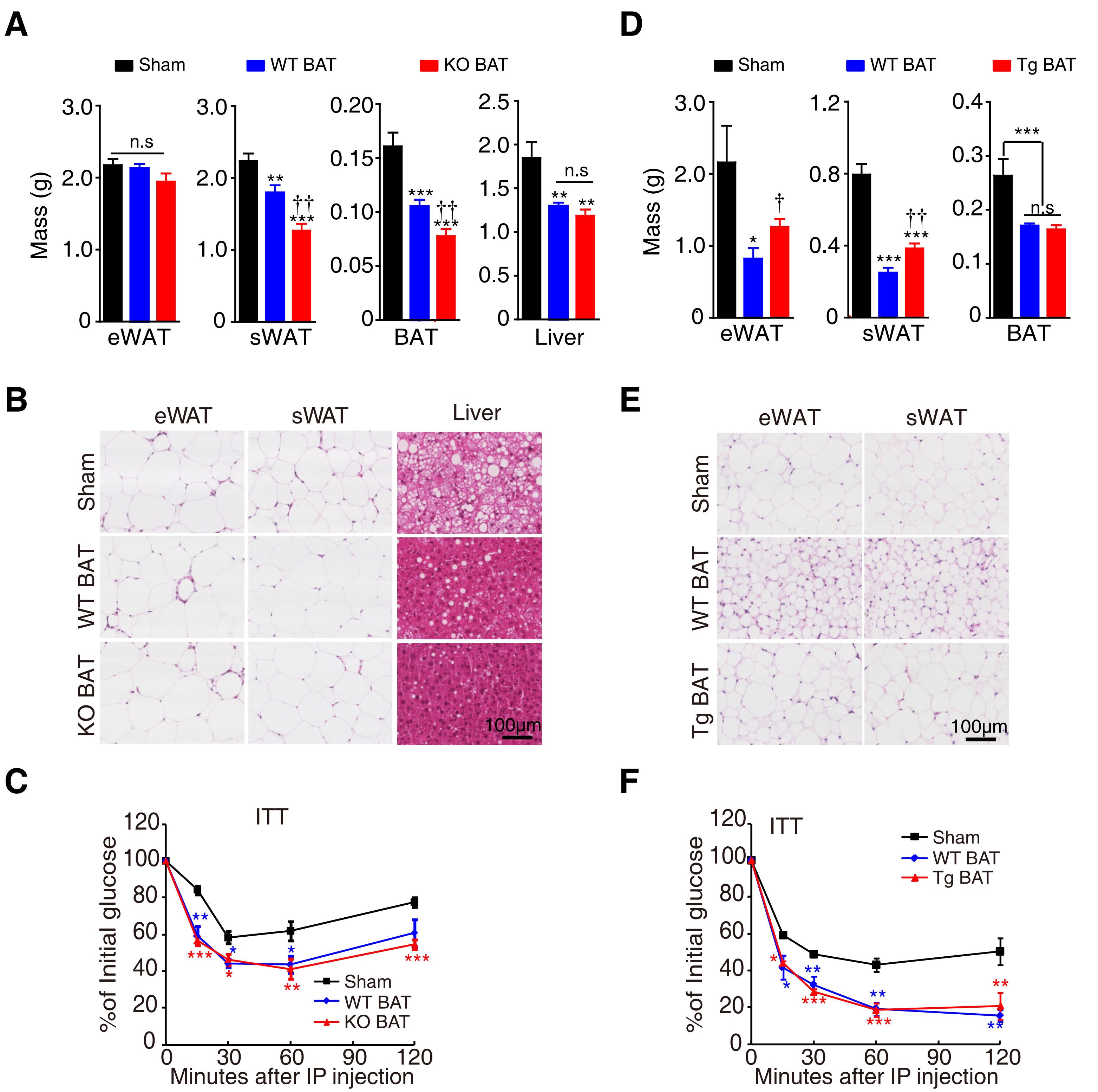
A
Sham
WT BAT
KO BAT
Mass (g)
n.s
eWAT
sWAT
BAT
Liver
B
eWAT
sWAT
Liver
Sham
WT BAT
KO BAT
100μm
C
ITT
%of Initial glucose
Minutes after IP injection
Sham
WT BAT
KO BAT
D
Sham
WT BAT
Tg BAT
Mass (g)
eWAT
sWAT
BAT
n.s
E
eWAT
sWAT
Sham
WT BAT
Tg BAT
100μm
F
ITT
%of Initial glucose
Minutes after IP injection
Sham
WT BAT
Tg BAT

Figure S6

A

Protein Name	Peptides
DJ-1	18
Mib2 (Isoform 2)	5
Smurf1	1

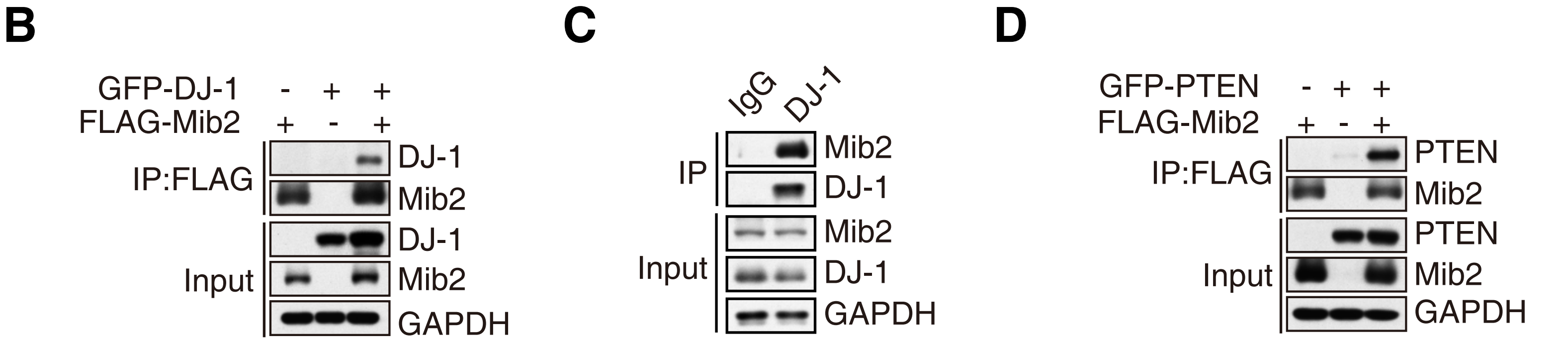

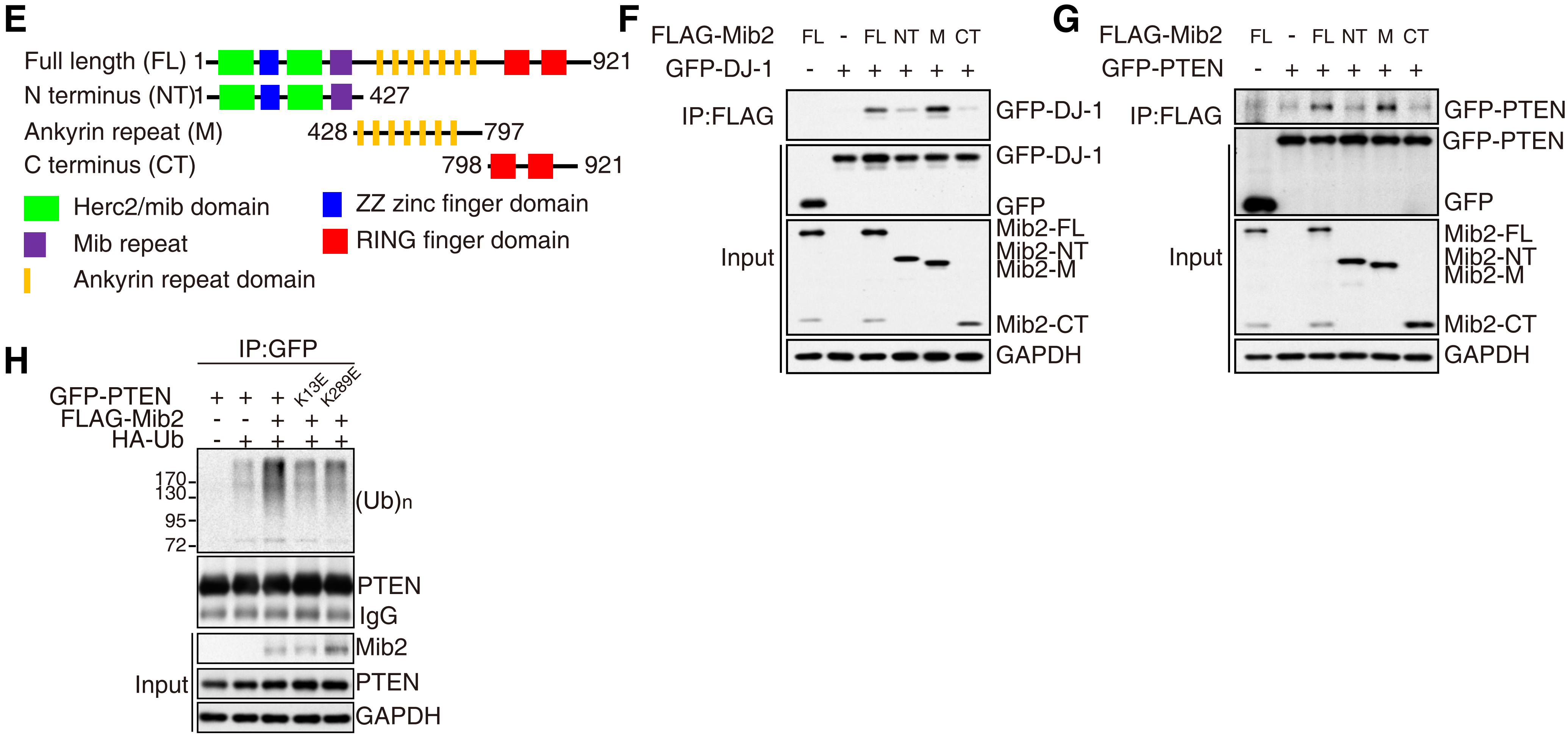

Figure S7

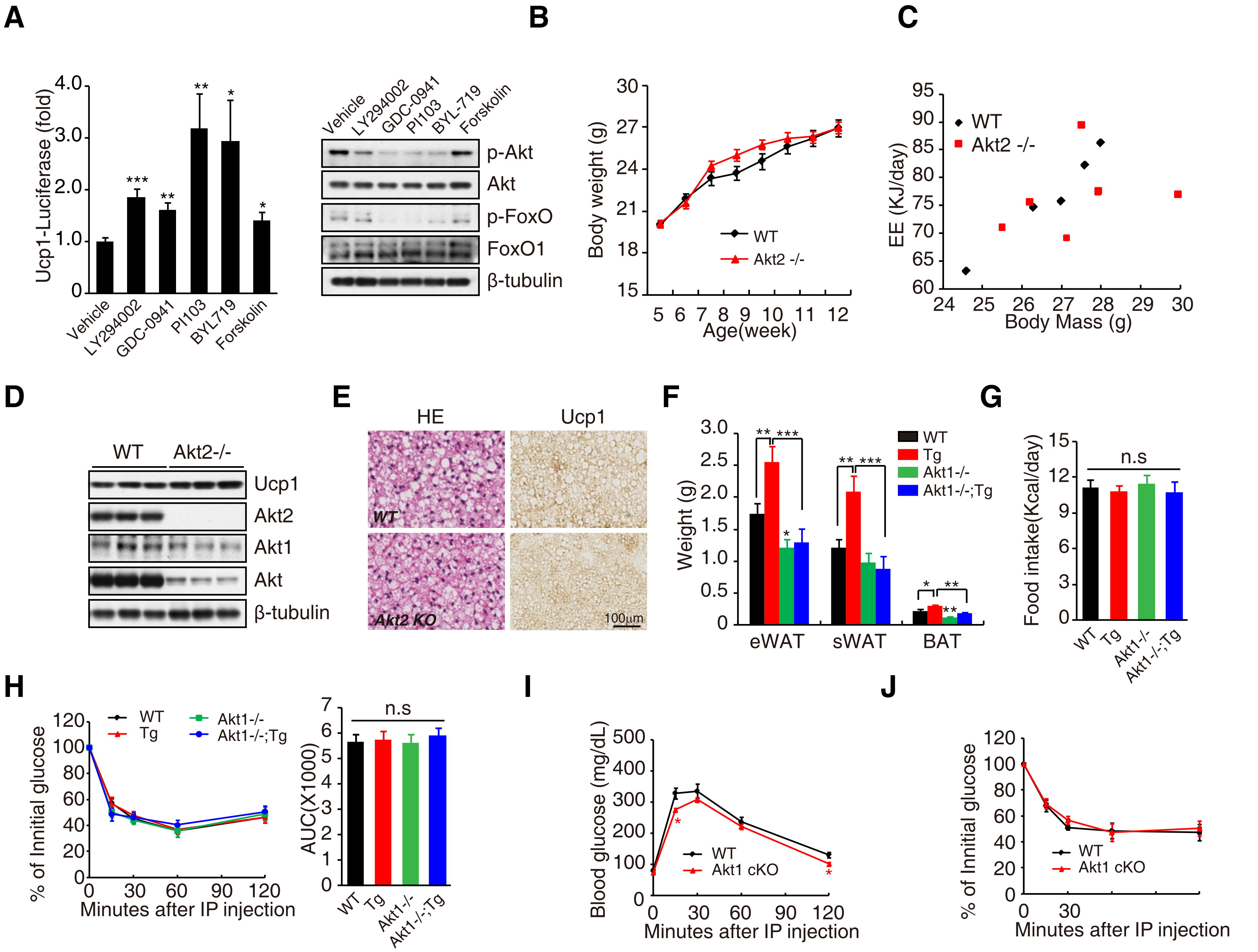

A
Ucp1-Luciferase (fold)
Vehicle
LY294002
GDC-0941
PI103
BYL719
Forskolin
p-Akt
Akt
p-FoxO
FoxO1
β-tubulin
B
Body weight (g)
Age(week)
WT
Akt2 -/-
C
EE (KJ/day)
Body Mass (g)
WT
Akt2 -/-
D
WT
Akt2-/-
Ucp1
Akt2
Akt1
Akt
β-tubulin
E
HE
Ucp1
WT
Akt2 KO
100μm
F
Weight (g)
eWAT
sWAT
BAT
WT
Tg
Akt1-/-
Akt1-/-;Tg
G
Food intake(Kcal/day)
n.s
WT
Tg
Akt1-/-
Akt1-/-;Tg
H
% of Innitial glucose
Minutes after IP injection
WT
Tg
Akt1-/-
Akt1-/-;Tg
AUC(X1000)
n.s
I
Blood glucose (mg/dL)
Minutes after IP injection
WT
Akt1 cKO
J
% of Innitial glucose
Minutes after IP injection
WT
Akt1 cKO
